# No Observed Adverse Effects on Health Were Detected in Adult Beagle Dogs When Fed a High-Calcium Diet for 40 Weeks

**DOI:** 10.3390/ani11061799

**Published:** 2021-06-16

**Authors:** Jujhar Atwal, Jonathan Stockman, Matthew Gilham, David Allaway, Helen Renfrew, Anne Marie Bakke, Phillip Watson

**Affiliations:** 1 WALTHAM Petcare Science Institute, Melton Mowbray, Leicestershire LE14 4RT, UK; jonathan.stockman@liu.edu (J.S.); matthewgilham96@googlemail.com (M.G.); david.allaway@effem.com (D.A.); anne.marie.bakke@effem.com (A.M.B.); phillip.watson@effem.com (P.W.); 2 Renfrew Imaging, Grove Road, Bladon, Woodstock OX20 1RD, UK; helen@renfrewimaging.co.uk

**Keywords:** canine, nutrition, phosphorus, calcium: phosphorus ratio, mineral balance

## Abstract

**Simple Summary:**

Calcium (Ca) is an essential mineral that plays a vital role in many bodily functions. There is evidence that high levels of dietary Ca for puppies and growing dogs can result in adverse growth and health effects, with certain breeds and dog size categories being most susceptible, for example, large breed sizes or beagles. Until recently, little was known about the health consequences of high Ca diets to adult dog, however, a study in the large sized breed Labrador retriever found no adverse health consequences when feeding diets containing levels of Ca near maximum levels set by pet food regulators (2.5 g per 100 g dry matter) for 40 weeks. This current study fed a similar high Ca diet to adult beagles, a medium sized breed for 40 weeks and monitored the dogs’ health using an extensive suite of health parameters. All dogs remained healthy and did not display any clinically relevant signs of adverse health relating to diet at any point during or following completion of the study.

**Abstract:**

The implications of long-term high calcium (Ca) intake are well documented in growing dogs and in adult dogs of large breed size, however, the consequences on other breeds and breed sizes are yet to be determined. Eighteen neutered adult beagles, nine males and nine females aged 1.4–4.4 years, were randomized to control or test diets providing in g∙4184 kJ^−1^ (1000 kcal^−1^): 1.44 and 7.19 total Ca balanced with 1.05 and 4.25 total phosphorus, respectively, for 40 weeks. Health parameters, ultrasound scans, radiographs, glomerular filtration rate, and mineral balance were measured at eight-week intervals. All dogs remained healthy with no measured evidence of orthopedic, urinary, or renal disease. The test diet resulted in a 5.2 fold increase in fecal Ca excretion. Apparent Ca digestibility (%) and Ca balance (g/d) did not significantly (*p* > 0.05) change from baseline in the test diet group, although dogs displayed a positive Ca balance (maximum at week 8, 1.11 g/d with 95% CI (0.41, 1.80)) before a neutral Ca balance was restored at week 32. Despite an initial positive Ca balance, we can conclude that no measurable adverse health effects were observed as a result of the test diet fed in this study in beagles over a period of 40 weeks.

## 1. Introduction

Calcium (Ca) is an essential mineral that is vital in many physiological functions including bone development, muscle contraction, nerve signal transmission, and blood coagulation. Maintaining Ca homeostasis is a complex process that involves bones, kidney, intestines, and the parathyroid gland in conjunction with hormones and hormone-like compounds including parathyroid hormone (PTH), calcitonin, fibroblast growth factor 23 (FGF-23), and vitamin D metabolites such as calcitriol (1,25-dihydroxyvitamin D (1,25(OH)_2_D)) and possibly dihydroxycholecalciferol (24,25-dihydroxyvitamin D_3_ (24,25(OH)_2_D_3_)). These precisely regulate intestinal absorption, renal excretion, and skeletal store mobilization [1,2].

From an evolutionary perspective, dogs are predatory and scavenging animals, and as such, their dietary intake would consist primarily of foodstuffs from carcasses including bones, which are abundant in Ca and phosphorus (P), among other minerals. Thus, it could be assumed that dogs have well developed regulatory mechanisms to manage variable, possibly often excess, mineral intake. However, studies have established that high levels of dietary Ca for puppies and growing dogs can result in adverse growth and health effects, with certain breeds and dog size categories being most susceptible [3,4,5,6]. Cofactors such as health status [7,8] or synergistic or inhibitory interactions of Ca with other minerals [9,10,11,12,13], particularly P and vitamins [14,15], are also thought to be influential in modifying its potential effect on health.

A meta-analysis representing 438 digestibility studies reported a linear relationship between Ca intake and Ca fecal excretion, proposing that adult dogs do not have the ability to regulate intestinal Ca absorption in response to dietary Ca intake [16]. The meta-analysis also reported that Ca and protein source or dog breed were not influential factors. The digestibility studies included in this meta-analysis were 3–28 days in length, median eight days, and therefore only limited insights on the long-term effects of feeding at set levels of Ca were gained. A study conducted with two breeds of adult dogs fed diets containing Ca at the National Research Council (NRC) minimum requirement (0.5 g/4184 kJ (1000 kcal)) [17] for 28 weeks support the conclusions drawn by the meta-analysis [16], providing further indication that dogs may not increase intestinal Ca absorption when dietary Ca is limited. Instead, the data showed an increase in a bone resorption marker, suggesting that mobilization of Ca from bone was preferentially employed to maintain Ca homeostasis [18], which has also been reported in other mammalian species [19]. A disruption to bone markers and serum Ca has also been reported when beagles were fed a low Ca diet for 14 days [20].

Breed or body size differences may affect Ca tolerance in growing dogs, as a study that investigated feeding a high Ca diet (3.6% dry matter (DM)) to beagle and foxhound-crossbred puppies demonstrated. Radiographs revealed reduced growth in radial and ulnar bones over a period of 21 weeks in beagles only. The larger foxhound crossbred dogs displayed no adverse clinical or subclinical effects to high dietary Ca [3]. This study may support the view that differences in breed susceptibility to high Ca exists in growing dogs. High Ca intake has also been linked to joint and urinary health, although all the contributing factors are not well understood [3,4,5,6,8,21,22,23,24].

The regulation of high dietary Ca in adult dogs is not well documented and extrapolated data from growth studies have been used in setting nutrient guidelines. The Association of American Feed Control Officials (AAFCO) [25] and European Pet Food Industry Federation (FEDIAF) [26] have set maxima of 2.5% DM Ca in diets intended for adult dogs, while maximum levels for growing and large size puppies are lower (1.6–1.8% DM Ca). The NRC [17] does not specify safe upper limits for Ca.

To provide further evidence of the tolerance for and regulation of high dietary Ca in adult dogs, and thus further confidence in the maximum Ca levels specified by AAFCO and FEDIAF [25,26], a series of investigations were initiated to establish long-term feeding safety. In contrast to the conclusions drawn by the meta-analysis [16], a study of the large breed Labrador retriever found that adult dogs were able to adapt intestinal Ca absorption when fed levels of dietary Ca at 2.61% DM (7.10 g/4184 kJ (1000 kcal)) over a period of 40 weeks [27], with no observed adverse health effects.

The primary purpose of the current study was to determine if an upper limit for Ca established as safe for the large sized breed, adult Labrador retriever is also safe for adult beagles over a period of 40 weeks; a medium sized breed previously reported to develop skeletal anomalies during growth when exposed to high dietary Ca levels [3]. The requirement to balance Ca with P levels to a desired Ca:P ratio between 1:1 and 2:1 [25,26] also provided a secondary opportunity to investigate the safety of the long-term feeding of P close to the published maximum levels. High levels of dietary P has been implicated with renal disease in several species [28,29,30].

## 2. Materials and Methods

### 2.1. Study Design

In a parallel study design, eighteen adult beagle dogs were randomized into one of two diet groups (control or test), balancing for sex, age, bodyweight (BW), familial affiliation (litter mates), and energy requirements to maintain ideal body condition score (BCS). The control group comprised eight dogs and the test group comprised ten dogs. Dogs were offered one of two dry format diets differing in Ca and P levels (control and test) for 40 weeks following a nine-week period where all dogs were fed the control diet and baseline measurements were collected. The research protocol was reviewed and approved by the WALTHAM Animal Welfare and Ethical Review Body and carried out under the authority of the Animals (Scientific Procedures) Act (1986).

### 2.2. Dogs and Housing

Healthy, neutered adult beagle dogs, nine males and nine females, from a total of nine litters, aged 1.4–4.4 years (mean 2.0 years; median 1.4 years) were selected for the study. The dogs were selected from a larger group following behavioral assessments and health-screening including assessment of blood hematology, biochemistry, and urinary health parameters as well as a physical health check conducted by a veterinarian. Only dogs with health parameters within normal ranges were considered for inclusion on the study. All dogs were housed in pairs according to diet group to minimize risk of feeding error and to prevent access to feces related to other diets. Dogs were housed in pens measuring between 7.3–7.6 m^2^ with solid floors, with direct access to outdoor runs measuring between 4.5 and 6.1 m^2^ between 8 am. and 4 pm. Dogs received one morning meal supplying 80% of maintenance energy requirement per day (MER) based on an amount to maintain a healthy BCS and reviewed weekly. Any meal refusal was reoffered to the dog in the afternoon. Dogs received the remaining 20% MER diet allowance during training and for treats throughout the day as positive reinforcement. Diet was offered in amounts to maintain an ideal BCS according to the WALTHAM Size, Health and Physical Evaluation guide (S.H.A.P.E) [31]. Deionized water was freely available to dogs at all times. During urine and feces collection, dogs were housed individually in pens measuring 13.7 m^2^ with solid floors and monitored continuously for 120 h to allow sample collection and to prevent coprophagia. During periods of individual housing, dogs had supervised socialization time with dogs within diet group for a minimum of one hour per day and increased socialization time with carers and trainers. All dogs were examined by a veterinarian at baseline and at eight-week intervals (8, 16, 24, 32, and 40 weeks), which consisted of BCS, heart rate, respiration rate, urogenital, dermatological, ears, eyes, nose, and throat assessments. Routine clinical biochemistry, hematology, and urinalysis parameters (see below) were also reviewed.

### 2.3. Diets

Single batches of two extruded dry format experimental diets were specifically formulated and manufactured for this study (Royal Canin, Aimargues, France). Control and test diets used the same core recipe, which were also used in a former study [27] containing similar amounts of pork meal, pork fat and rind, fish and soya oil, cereals, beet pulp, flavorings, vitamin, and antioxidant mixes. Control and test diets targeted minimum maintenance and maximum Ca and P levels, respectively [25,26]. Control diet contained 1.44 g total Ca/4184 kJ (1000 kcal) and 1.05 g total P/4184 kJ (1000 kcal) (Ca:P 1.4), whereas the test diet contained 7.19 g total Ca/4184 kJ (1000 kcal) and 4.25 g total P/4184 kJ (1000 kcal) (Ca:P 1.7). The total Ca and P levels corresponded to 144 and 719% of the recommended Ca allowance and 133 and 573% of the recommended P allowance for adult dogs according to NRC [17], for the control and test diets, respectively. The additional Ca and P content of the test diet was achieved by supplementation with inorganic sources: Ca carbonate (CaCO_3_), monocalcium phosphate (Ca(H₂PO₄)₂.H₂O; MCP), and Ca sulfate (CaSO_4_). CaCO_3_, MCP, and CaSO_4_ provided 48%, 40%, and 6% of the Ca, respectively, and MCP provided 88% of the P in the test diet. CaCO3 provided 56% of the Ca in the control diet, which contained no MCP or CaSO_4_. A small amount of inorganic P (0.10 g/4184 kJ (1000 kcal)) was included in both diets as a palatant and a carrier of a vitamin premix; this provided 10% and 2% of the P to the control and test diet, respectively. The nutrient composition analysis of both diets was completed at the Mars Petcare Europe Central Laboratory (Aimargues, France) and Eurofins Ltd. (Wolverhampton, United Kingdom) post production (Table 1; Table S1). Average post manufacture diet analysis values revealed differences between the control and test diet exceeding 20% in Ca, copper, docosahexenoic acid, eicosapentenoic acid, iron, magnesium, manganese, P, protein, riboflavin, thiamin, valine, vitamin E, and zinc. The Ca:P differed between the control and test diets, however, were within the desirable range [16,25,26]. The increased dietary Ca and P required for the test diet resulted in a 54% increase in dietary ash, which consequently resulted in a 20% reduction in protein compared to the control diet. Other differences between diets or from specification targets may be due to variability of raw ingredients during manufacture. With the exception of Ca, P, and riboflavin (vitamin B2) in the test diet, both diets were compliant with the AAFCO [32] and NRC [17] recommended allowance for adult dogs. Weekly riboflavin supplementation (SOLGB73050, Solgar Inc., Hertfordshire, United Kingdom) was added to the test diet to provide a total of 1.8 mg/4184 kJ (1000 kcal).

### 2.4. Measures

Blood and urinary health parameter analysis, glomerular filtration rate (GFR), diet digestibility, mineral balance, urine relative super saturation (RSS), radiography, and abdominal ultrasound scans were performed at baseline and at eight-week intervals (8, 16, 24, 32, and 40 weeks). Dual-energy x-ray absorptiometry (DXA) was performed at baseline and at 24 and 40 weeks. On very few occasions, due to behavioral or resource availability reasons, some measures were collected within a maximum of two weeks from the planned sample collection.

### 2.5. Intake, BW, and BCS

Diet intake was recorded daily per individual dog as mass (g) of diet offered minus mass (g) of diet refused. BW was recorded weekly (kg) on a digital weighing platform (PBD655-CC60, Mettler Toledo, Columbus, OH, USA). BCS was recorded weekly by trained scorers using the WALTHAM S.H.A.P.E guide [31].

### 2.6. Mineral Balance and Apparent Digestibility

All feces excreted by the dogs were collected over five total consecutive days (120 h) at each time point. Feces were frozen and stored at –20 °C in a sealed container per individual dog prior to processing. Each pooled five day sample per individual dog was weighed and freeze-dried (One VirTis BenchTop BTP9ES Freeze Dryer; Biopharma Process Systems, Winchester, United Kingdom). Once dried, the feces were weighed again and manually homogenized with a mortar and pestle. Any foreign materials such as stones and plant material were removed and weighed separately.

A 50 g sample of the homogenate was analyzed for moisture, fiber, and ash according to published methods [35]. Fat was determined by acid hydrolysis followed by diethyl ether extraction and protein was determined from total N content by combustion using the Dumas principle according to Association Française de Normalisation (AFNOR ISO 16634-1:2008 November 2008). Ca was determined by flame photometry and P by spectral photometry according to AFNOR methodology (Invivo Labs, Saint-Nolff, France).

Apparent digestibilities of protein, fat, DM, Ca, and P were calculated as the difference between the dietary intake and fecal excretion content over the sample collection days and expressed as percentage of the intake; Apparent digestibility (%) = ((intake — excreted fecal excretion)/intake) × 100.

Mineral balance (g) was calculated for Ca and phosphate as total mineral balance (g) = intake — (fecal excretion + urinary excretion).

A positive apparent digestibility indicates feces losses in proportion or less than intake whilst a negative apparent digestibility indicates fecal loss exceeds intake. Mineral balance includes urinary excretion values.

### 2.7. Urine Mineral Content and RSS

Urine was collected over three consecutive days (72 h) using a free-catch method at each time point, collecting on average 79% of all urine excreted from the dogs. Missed urine collections and spillages were estimated by recording urination duration or approximating urine puddle size; we estimated this accounted for 15% and 6% of the total volume excreted, respectively. All urine collected was pooled per individual dog prior to twice daily pH assessment to detect for bacterial contamination. Urine determined free from contamination was transferred to a three day pool bottle that contained a drop of chlorohexidine (C9394; Sigma Aldrich, St. Louis, MO, USA) to prevent spoilage. After the collection period, an aliquot of urine was acidified to pH 2 with the addition of 36.5–38.0% hydrochloric acid (H1758; Sigma Aldrich). The method for RSS analysis has been previously described [36], samples were analyzed by high performance liquid chromatography for ammonium, Ca, citrate, creatinine, magnesium, oxalate, phosphate, potassium, sodium, sulfate, and uric acid (Mars Europe Petcare Central Laboratory, Aimargues, France). The concentrations of analytes were used by SUPERSAT [36] software to calculate the RSS (activity product/solubility product) for struvite (magnesium ammonium phosphate (MAP)) and Ca oxalate (CaOx). The concentrations of Ca and phosphate measured in urine were used to calculate the urinary fractional excretion of these minerals.

### 2.8. Urinalysis

A 5 mL freshly voided urine sample was collected from each dog using a free-catch method at each time point and analyzed semi-quantitatively for bilirubin, blood, glucose, ketone, leukocyte, nitrite, pH, protein, and urobilinogen (Siemens Multistix 10SG; Siemens AG, Erlangen, Germany) within 30 min. Specific gravity was determined by refractometer (Sinotech RHCN-200ATC; Sinotech, Zhangzhou, China). Urine albumin was quantified using a canine specific ELISA (ab157695; Abcam PLC, Cambridge, United Kingdom) and urine creatinine was quantified (OSR6178; Beckman Coulter, Brea, CA, USA) via Olympus AU480 Biochemistry analyzer (Olympus Europe GmbH, Hamburg, Germany). The urine albumin:creatinine ratio (UACR) was calculated (mg/g) for each sample.

### 2.9. Blood Based Measures

A total of 8 mL of fasted blood (at least 12 h since last meal) was drawn from the jugular vein at each time point. For behavioral reasons, blood was collected from a lateral saphenous vein via catheter for one dog on one occasion (week 16). Blood was decanted into five tubes (1 × 0.2 mL EDTA, 1 × 0.5 mL lithium heparin, 1 × 0.3 mL lithium heparin and 2 × 4.4 mL gel-activated serum clot). EDTA (0.2 mL) treated whole blood was assessed for complete blood counts (leucocyte and erythrocyte counts, hemoglobin concentration, hematocrit, platelet count, mean corpuscular volume, mean corpuscular hemoglobin, lymphocyte count, monocyte count and granulocyte count) using a hematology analyzer (Mythic 18; Orphée SA, Plan-les-Ouates, Switzerland). Plasma was separated from lithium heparinized blood (0.5 mL) via centrifugation (1999 g for ten minutes at 4 °C) and was analyzed to measure the biochemical parameters (alanine transaminase, albumin, alkaline phosphatase, aspartate aminotransferase, Ca, chloride, cholesterol, creatinine, glucose, inorganic P, potassium, sodium, total protein, triglycerides, and urea) using a biochemistry analyzer (Olympus AU480; Olympus Europe GmbH, Hamburg, Germany). Lithium heparinized whole blood (0.3 mL) was used to quantify ionized Ca (iCa) (Stat Profile Prime Critical Care Analyzer; Nova Biomedical, Waltham, MA, USA). Serum was separated from blood in gel-activated serum clot tubes by centrifugation (1999 g for ten minutes at room temperature). Serum was fractioned into different storage tubes and stored at –80 °C prior to the analysis of PTH, FGF-23, vitamin D metabolites, serum cross-laps (C-terminal telopeptide (CTx)), and bone alkaline phosphatase (BAP). PTH and FGF-23 analysis was carried out at the Royal Veterinary College, United Kingdom. PTH concentrations were quantified using a human total intact PTH immunoradiometric assay (Scantibodies Laboratory Inc., Santee, CA, USA) validated and previously used with canine samples [27,37,38,39]. FGF-23 concentrations were quantified using a human sandwich ELISA (Kainos Laboratories Inc., Tokyo, Japan) validated and previously used with canine samples [27,40]. Analysis of vitamin D metabolites were conducted at the bioanalytical facility at the University of East Anglia, United Kingdom using validated methods [41]. Vitamin D metabolites 25OHD (total calcidiol), 24,25(OH)_2_D_3_ (24,25-dihydroxycholecalciferol), 24,25(OH)D_2_ (24,25-dihydroxyergocalciferol), total 24,25(OH)_2_D (total dihydroxycalcidiol), 25OHD_3_ (calcidiol), and 25OHD_2_ (ergocalcidiol) were measured using non-epimer resolved LC/MS-MS (performed using a Micromass Quattro Ultima Pt Mass Spectrometer (Waters Corp., Milford, MA, USA). Total 1,25(OH)_2_D (calcitriol) was measured using an EIA Kit (IDS, Boldon, UK). BAP and CTx were analyzed using validated enzyme-immunoassays (MicroVue Bone Health BAP EIA Kit (Quidel, San Diego, CA, USA) and CTx-1 ELISA (IDS, Boldon, UK), respectively). 

### 2.10. GFR

GFR was estimated using the iohexol clearance method [42] on fasted dogs at each time point. Iohexol (647 mg/kg; Omnipaque 300, Amersham Health, Princeton, NJ, USA) containing 300 mg/kg iodine was administered over a three minute time period followed by a 0.5 mL heparinized saline flush into the lateral saphenous or cephalic vein via catheter. The completion of the injection represented time zero. 2 mL blood samples were collected from the cephalic vein via catheter at 2, 3, and 4 h post iohexol infusion and decanted into blood tubes (2 × 1.1 mL gel-activated serum clot) at each time point. Serum was separated from blood by centrifugation (1999 g for ten minutes at room temperature) and stored at –80 °C prior to analysis. Iohexol concentration in the serum of each dog at each time point was analyzed using high-performance capillary electrophoresis by deltaDOT Ltd., United Kingdom. Weight adjusted clearance (ml/kg per min) was calculated using the slope of the concentration gradient over the three time points. 

### 2.11. Imaging

Ultrasound imaging and radiography was completed on fasted, sedated dogs to detect any soft tissue mineralization, urolithiasis, or other pathologies. Ventrodorsal and right lateral recumbent abdominal radiographs, right lateral recumbent thoracic radiographs (AGFA CR 30-X, exposure 46–64 kV, 2·36–4·73 mAs; AGFA) and full abdominal ultrasound scans (GE Logiq-E with microconvex probe (8 C), 4·0–10·0 MHz) were completed at each time point. Sedation was induced through administration of butorphanol (250 µg/kg) and medetomidine (10 µg/kg) and reversed through the administration of atipamazole (50 µg/kg). Interpretation of ultrasound scans and radiographs were carried out by a diplomate of the European College of Veterinary Diagnostic Imaging (H. R.) who was blinded to the dog diet group. At baseline, 24, and 40 weeks, DXA (EncoreTM 2006, 10.50 software; GE Lunar Prodigy Advance) scans were also performed whilst dogs were sedated in the supine position to determine bone mineral density (BMD; g/cm^2^) and bone mineral content (BMC; g). Briefly, whole body scans were performed, which took 4 min and 30 s (0.0003 mGy (<1%CV); kV 76). Total body BMD was determined combining BMD for the head, arms, legs, rib, pelvis, and spine region. DXA has been previously validated and used in dogs [27,43,44]. Quality assurance and calibration were performed daily on the day of use and monthly when not in use using the manufacturer’s anthropomorphic spine phantom and quality control software.

### 2.12. Statistical Powering

Study powering was performed for the primary response variable, percentage difference in intake, and excreted fecal Ca levels (mg/kg^0.75^) by simulation using data from a previous study [18]. Results indicated that to detect a 6% reduction from a baseline of 12% in Ca retention, with at least 80% power, ten dogs were required in the test group and eight in the control group.

### 2.13. Statistical Analysis

The data were analyzed using linear mixed effects models with each measure as the response. Log10 transforms were applied to several measures, consistent with the analysis of data in the former study [27]. Fixed effects of diet, sampling occasion, and their interaction, and a random effect of dog to account for the repeat measurements were included in these models. Data consisting of duplicate measurements per dog, per sampling occasion, had a modified random effect with sample nested in dog. Three sets of hypothesis tests were performed; first, the difference between diet groups at each of the sampling occasion time points, second, the change from baseline to each time point within each diet group; and finally, the difference in the change from baseline between diet groups. For the mineral balance measures, the estimated means by diet group and time point were tested for differences versus zero. Ca intake and fecal excretion data were log10 transformed and compared using a linear mixed effects model. This model had Ca fecal excretion as the response and the interaction between Ca intake and diet as the regressors. A random effect of dog to account for repeated measurements was also used. With this model, the intake versus excretion relationship, with 95% confidence intervals (CI), was estimated for both diets and visualized on a single figure, alongside a 1:1 line. In total, 1.7% of data were missing or excluded from analysis due to not meeting the quality control criteria (1.2%), sample contamination (0.4%), or results being below the detection limit of the assay (0.1%). All analyses were performed using R v3.5.1 software with the lme4 and multcomp libraries [45,46,47] and the hypothesis tests were corrected for multiplicity, separately for each outcome measure, to control the family-wise error rate to a level of 5%. Data are presented as means with 95% CI.

## 3. Results

### 3.1. General

All eighteen dogs completed the full duration of the study. Health status was determined based on a veterinarian review of the blood biochemistry, hematology, urinalysis, and physical health exam results completed every eight weeks and any other health concerns reported during the study. All dogs remained healthy and did not display any clinically relevant signs of adverse health relating to diet intake at any point during or following completion of the study. With the exception of plasma urea, no consistent differences in biochemistry and hematology health measures were found that could be attributed to the diets fed in this study (Tables S2 and S3).

### 3.2. Intake and BW

Both control and test diets were well accepted by the dogs throughout the study. Energy intake was not significantly different between the diet groups at any time point, although it declined over time, resulting in a significant (*p* < 0.05) difference from the baseline in both diet groups at week 32 and 40 (Table 2). Ca and P intake increased after baseline for the test group dogs once they transitioned to the test diet (Table 2). BW was not significantly different between diet groups or from baseline in either diet group at any time point (Table 2). Instances of coprophagia were low, three reports of observed or suspected coprophagia were recorded during the study, and each occasion involved the same two test group dogs consuming a small amount of feces.

### 3.3. Ca, P, Regulatory Hormones and Vitamin D Metabolites in Blood

Plasma Ca and P levels were not significantly different between the diet groups at any time point. Plasma Ca increased over time, resulting in a significant (*p* < 0.05) difference from the baseline in both diet groups at week 24, 32, and 40. Plasma P significantly (*p* < 0.05) decreased from the baseline in the test diet group at week 32 and 40, however, the values remained within the normal reference range (0.8–1.60 mmol/L) (Table 3). iCa measured in the test group was not significantly different from the baseline at any time point during the study. A significant (*p* < 0.001) difference between diet groups was found at week 8 due to a significant (*p* < 0.001) drop of iCa at week 8 from the baseline in the control group, however, values remained within the normal reference range (1.12–1.40 mmol/L) (Table 3). PTH, FGF-23, and the vitamin D metabolite total calcidiol were not significantly different between the diet groups at any time point, nor did calcitriol significantly change from the baseline in the test group at any time point during the study; in contrast, calcitriol in the control group significantly (*p* = 0.004) declined from the baseline at week 8 and remained lower at all subsequent time points (Table 3). Calcitriol data for nine samples were below the assay’s limit of detection (<12 pmol/L) and thus not included in the analysis; eight from the control group (one at baseline, two at week 8, two at week 16, one at weeks 24, 32 and 40) and one from the test group at week 32. No significant differences between diet group were found in any of the other vitamin D metabolites measured including 24,25(OH)_2_D_3_, 24,25(OH)D_2_, 24,25(OH)_2_D, 25OHD_3_, and 25OHD_2_, although significant (*p* < 0.05) changes over time were observed for both diet groups for all of these metabolites with the exception of 25OHD_3_. As a result, the ratio between total calcidiol and total dihydroxycalcidiol (25OHD:24,25(OH)_2_D) significantly (*p* < 0.05) increased in both diet groups compared to the baseline during the course of the study (Table 3).

### 3.4. Diet Digestibility and Mineral Balance

**Figure 1:**
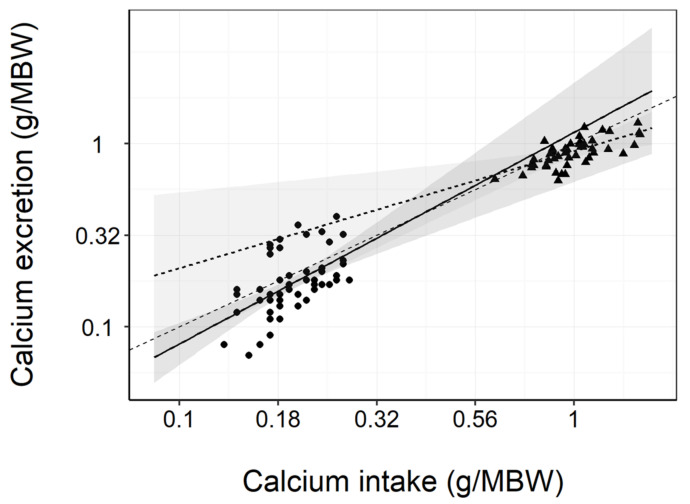
Linear regression between dietary calcium (Ca) intake and fecal Ca excretion (g/kg bodyweight (BW)^0.75^; metabolic bodyweight (MBW)). The 95% confidence regions for both diet groups surround the y = x (-------) equivalence line. The control (*n* = 56; —●—) and test (*n* = 50; ---▲---) display as distinct clusters supporting the view that Ca excretion is in proportion to dietary Ca intake.

Fecal Ca and P excretion were not significantly different between diet groups at baseline. On average, a 5.2 and 5.6 fold significant (*p* < 0.001) increase was found in fecal Ca and P excretion, respectively, once the test group dogs transitioned onto the test diet and this response remained throughout the study. A significant (*p* < 0.001) increase in fecal Ca and P excretion was also found in the control group at week 8, however, this reverted back to a level not significantly different from the baseline at all subsequent time points (Table S4). Fecal Ca excretion was found to be approximate to Ca intake (Figure 1).

**Figure 2:**
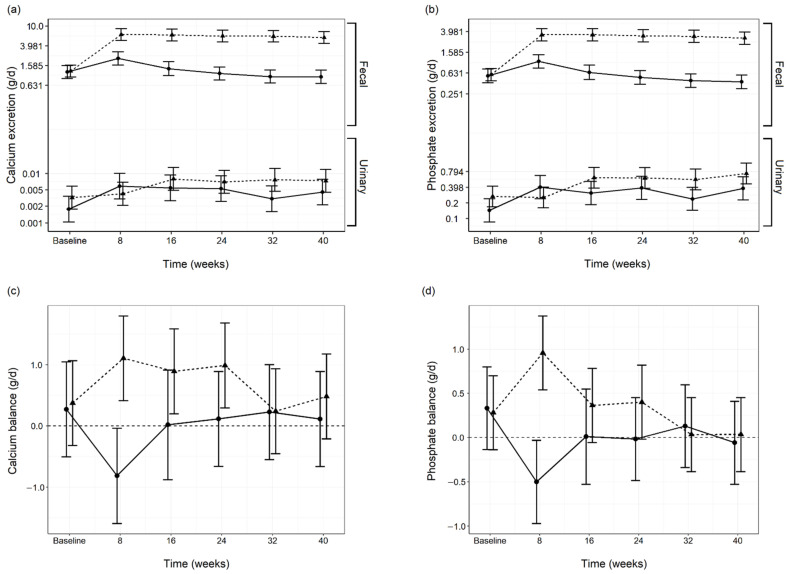
Fecal and urinary calcium (Ca) excretion (**a**), fecal and urinary phosphate excretion (**b**), Ca balance (**c**) and phosphate balance (**d**) throughout the study in both the control (*n =* 6–8; —●—) and test *(n =* 10; ---▲---) diet groups. Data values are presented as means and 95% confidence intervals per diet group per time point.

Urine Ca and phosphate losses significantly (*p* < 0.05) increased from the baseline in both diet groups. A significant (*p* < 0.05) between diet group difference was determined for both minerals at the 32 week time point due to a decline in urinary Ca and P excretion in the control group. However no significant between diet differences were determined at any other time point (Figure 2a,b; Table S4).

Apparent digestibility of Ca (Control 19.75 (−13.16, 52.66) %; Test 23.94 (−5.50, 53.37) %; *p* = 1.000) and P (Control 31.66 (14.69, 48.64) %; (Test 24.29 (9.11, 39.48) %; *p* = 0.991) were not significantly different between diet groups at the baseline. With the exception of a between diet group difference in apparent Ca digestibility at week 8 due to a significant (*p* < 0.001) decline in the control group, no other significant differences from the baseline or between diet group were determined. A significant (*p* < 0.001) between diet group difference in apparent P digestibility was determined at week 8 due to a significant (*p* < 0.001) decline in the control group. Significant (*p* < 0.05) changes from the baseline were found in both diet groups at other time points, however, no other significant differences between diet groups were determined (Table S4).

No significant differences in Ca (Control 0.27 (−0.51, 1.05) g/d; Test 0.37 (−0.32, 1.07) g/d; *p* = 1.000) and phosphate (Control 0.33 (−0.14, 0.80) g/d; (Test 0.28 (−0.14, 0.70) g/d; *p* = 1.000) balance were found between diet groups at the baseline. A significant difference in Ca (Control −0.81 (−1.59, −0.04) g/d; Test 1.11 (0.41, 1.80) g/d; *p* < 0.001) and phosphate (Control −0.50 (−0.97, −0.03) g/d; Test 0.96 (0.54, 1.38) g/d; *p* < 0.001) balance was found between diet group at week 8 due to a decrease in the control group and increase in the test group for both minerals. Otherwise Ca and phosphate balance did not significantly differ between diet group or from the baseline at any of the following time points (Figure 2c,d; Table S4). However, test group dogs consuming the test diet presented a significant (*p* < 0.05) positive Ca balance at the 8, 16, and 24 week time points, whereas the control group demonstrated a significant (*p* = 0.032) negative Ca balance at week 8 (Figure 2c; Table S4). The test group demonstrated a significant (*p* < 0.001) positive phosphate balance at week 8 whereas the control group demonstrated a significant (*p* = 0.027) negative phosphate balance. Phosphate balance was not significantly different from zero at any of the following time points for either diet group (Figure 2d; Table S4).

No significant differences in energy (Control 87.88 (85.63, 90.12) %; Test 88.82 (86.81, 90.83) %; *p* = 0.993), protein (Control 84.99 (82.23, 87.74) %; Test 86.47 (84.01, 88.93) %; *p* = 0.959), or fat (Control 95.55 (94.56, 96.54) %; Test 95.88 (94.99, 96.77) %; *p* = 0.999) digestibilities were determined between diet group at baseline and no sequential differences between diet groups or persistent changes over time were apparent. DM digestibility in the test group was significantly (*p* < 0.001) reduced at week 8 and remained reduced for the duration of the study whilst the control group did not significantly change over time (Control _Baseline_ 88.39 (85.81, 90.96) %, Control _End_ 87.90 (85.32, 90.48) %; Test _Baseline_ 89.21 (86.91, 91.51) %, Test _End_ 81.70 (79.40, 84.00)) (Table S4).

### 3.5. Renal and Urinary Health

**Figure 3:**
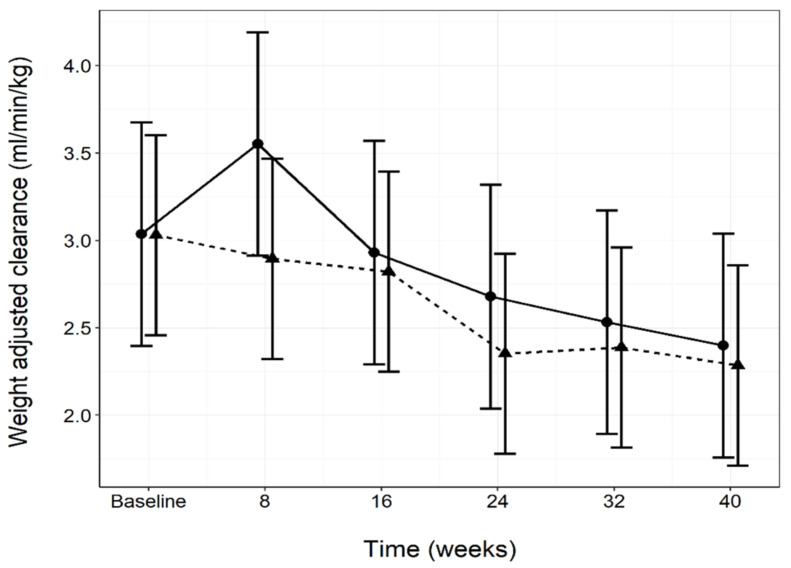
Glomerular filtration rate estimated by iohexol clearance, measured throughout the study in both the control (*n =* 8; —●—) and test (*n =* 10; ---▲---) diet groups. Data values are presented as means per diet group per time point and 95% confidence intervals.

No significant between diet group differences were determined in plasma creatinine or GFR (Figure 3; Table 4) during the course of the study. A significant (*p* < 0.05) increase in plasma creatinine compared to the baseline was observed in both diet groups, however, the values remained within the normal reference range (20–144.5 µmol/L) (Table 4). A significant (*p* < 0.05) decline in GFR from the baseline was observed in the test group at weeks 24 and 40 (Table 4). Plasma urea was significantly (*p* = 0.028) higher in the test group than the control group at the baseline, however declined whilst within the normal reference range (3.1–10.1 mmol/L) and remained significantly (*p* < 0.05) lower than the baseline for the remainder of the study. Significant (*p* < 0.05) increases in plasma urea in the control group from baseline at weeks 16 and 32 were found (Table 4). Specific gravity and UACR were not significantly different between diet groups at any time point. Although some significant (*p* < 0.05) differences from the baseline were found within diet group, these were not consistent over time (Table 4). RSS for CaOx in the test group was significantly (*p* = 0.001) higher than the control group at the baseline, but this decreased significantly (*p* = 0.003) at the following time point and remained stable for the remainder of the study. RSS for MAP was significantly (*p* < 0.001) different between the diet groups at week 8 due to a significant (*p* < 0.001) drop from the baseline in the test group. No other between diet differences at any time point were found (Table 4).

### 3.6. Bone Mineralization and Related Marker Measurements

DXA measurements of BMD (g/cm^2^) and BMC (g), and the bone formation marker BAP and bone resorption marker CTx were not significantly different between diet groups at any time point. BAP and CTx values significantly (*p* < 0.05) decreased from the baseline over time in both diet groups (Table 5).

### 3.7. Imaging

Thoracic and abdominal radiographic and ultrasonographic examinations performed at the baseline sampling revealed a number of irregular findings. One test group dog had soft tissue mineralization dorsal to the first sternebra, which was consistently present on radiographs throughout the study. Ultrasound scans found persistent presence of a fine renal rim sign with consistent echogenicity in eight dogs (three from the control and five from the test group) and pancreatic abnormalities in three dogs (two from the control and one from the test group). These findings were present at baseline and at every time point throughout the study, and did not change. Therefore, they were not considered as a consequence of the experimental diets, otherwise no evidence of soft tissue mineralization, calculi, urolithiasis, or skeletal changes were reported in any other dog at any point during the study.

## 4. Discussion

The primary purpose of this study was to determine whether a high dietary Ca intake, previously reported to not adversely affect health in large breed size adult dogs, was also safe when fed to a medium size breed adult dog over a period of 40 weeks. In addition, the requirement to balance Ca:P provided a secondary opportunity to investigate the consequences of long-term feeding of P close to the published maximum levels. Similar to the diets fed in the previous study undertaken with Labrador retrievers [27], the current study’s test diet levels of Ca and P were 13 and 5% higher, respectively, than the maximum specifications defined by AAFCO and FEDIAF [26,32]. Diets in both the current study and the Labrador retriever study were offered to dogs in amounts to maintain an ideal BCS and adjusted on a weekly basis. Consequently, beagles had approximately 50% higher energy intake at baseline when compared to Labrador retrievers [27], and subsequently higher Ca intake per kg/BW^0.75^. Many factors account for a difference in energy requirements (e.g., body size, breed, age, activity level, coat characteristics, and differences in ambient temperature). To minimize variability of environmental factors, the current study commenced 14 months after the Labrador retriever [27] study started, both studies occurred over winter and were completed during the summer season. Dogs in both studies had a similar daily routine consisting of similar levels of care, training, and recreation. The amount of diet offered to dogs in both diet groups in the current study was reduced over time to maintain ideal BCS, which consequently caused a reduction in mineral exposure during the course of the study. This resulted in a 20% decline in Ca intake between week 8 and 40 for the test group dogs. Ca intake (per kg BW^0.75^) at week 40 in the current study was more similar to the Ca intake (per kg BW^0.75^) in the test group during the Labrador retriever study (881 mg/kg BW^0.75^), where diet intake remained stable for both diet groups throughout the study [27].

A previous study demonstrated that large breed size dogs fed a high Ca diet regulated Ca balance by increasing fecal Ca excretion [27], which is consistent with the understanding that intestinal Ca absorption is the primary mechanism in Ca homeostasis in mammals [1,48]. Beagles in the test diet group had increased fecal excretion of Ca and P, however, urinary Ca and phosphate losses were not consistently different between diet groups and were negligible in comparison to amounts excreted in the feces. With the exception of week 8, apparent Ca and P digestibilities were not different between diet group, however, beagles in the test group displayed a positive Ca balance for at least 24 weeks and a positive phosphate balance for eight weeks before returning back to a level no longer significantly different from zero. One possible explanation is that beagles may undergo a period of adaptation to reach a neutral Ca balance, where Ca absorption is adjusted sufficiently to a point where Ca fecal excretion approximates Ca intake, thereby preventing unrestricted Ca accumulation. This would differ from findings in Labrador retriever [27], where no such adaptation period was observed. However, it is possible that Labrador retrievers have an adaptation period shorter than eight weeks, which would not have been identified with the trial design employed for the study. This explanation would need to be supported by further investigation in studies supplying a steady mineral intake. Alternatively, the positive mineral balance observed in the current study test group may simply be reflective of the higher intake of these minerals per kg BW^0.75^ when dogs initially transitioned to the test diet.

The temporary retention of small quantities of these minerals before a neutral balance was later restored did not manifest in any measurable adverse health or skeletal changes in the adult dogs during the study or following completion. The findings of the current study demonstrate that adult beagle dogs also tolerate and may regulate Ca homeostasis without adverse health consequences when provided with similar dietary levels as the previous study [27], despite differing mineral intake per unit BW and a positive Ca balance for the first six months of the study.

Health-related biochemistry and hematology parameters corroborate the view that the test diet had no observed adverse effect on systemic health. The decline in plasma urea found in the test group dogs once they transitioned to the test diet is likely due to the difference in protein content between the diets (Table 1). Plasma urea was not affected by diet in the Labrador retriever study [27], where protein content between diets was more similar (Control 29.9 g/100 g; Test 27.2 g/100 g). The decline from baseline in plasma P observed in the test diet group at the last two timepoints is unlikely to be of concern as values did not fall outside the reference range for a healthy adult dog. Test group values were not different to the control group at any time and were effectively the same at weeks 8 and 40.

Feeding the test diet for 40 weeks had no effect on plasma Ca or blood iCa concentrations. Dietary Ca intakes in excess of requirement could increase calcitonin and suppress synthesis of PTH from the chief cells of the parathyroid gland, which would subsequently suppress the activation of calcitriol from calcidiol in the kidney through the action of 1-α-hydroxylase [1]. Reduced level of calcitriol would limit the expression of Ca binding proteins and CaT1 that would result in reduced transcellular intestinal absorption capacity [48]. However, no statistically significant changes in PTH, total calcidiol, calcitriol, or other vitamin D metabolites in response to the test diet were observed, suggesting that either the Ca in the test diet was not at a level that would require hormonal regulation or this response occurred soon after transition to the test diet and before the 8-week sampling occasion. Recent findings indicate that feeding a diet containing high mineral levels from highly soluble inorganic sources, especially P, can result in a post-prandial increase in PTH in both cats [49] and dogs [50]. Blood sample collection in the current study was completed at the same time each morning on dogs fasted overnight as diurnal variations in some blood analytes such as PTH or P and bone markers would be expected [51,52], therefore it cannot be ruled out that post-prandial hormonal regulation may have differed between diet group.

The temporary decline in iCa in the control group at week 8 appears to have been driven by individual results of samples analyzed on a single day (*n =* 6) rather than being representative of all dogs in the control group (*n =* 8) at this time point. The reason for this transient decline is unclear, however, the absence of a decline in total plasma Ca or increases in PTH or calcitriol were not observed, as may be expected given the current understanding of mammalian Ca regulation [1]. Calcitriol declined after baseline measurements in the control group and this response was maintained for the remainder of the study. As the Ca levels in the control diet exceeded recommended allowance [17] and vitamin D levels were similar between diets (Table 1), this decline is likely to be due to other factor(s). In any case, these unexpected results in the control group do not impede the interpretation of the impact of feeding the test diet in this study.

It has been reported that feeding a diet containing 3.6% DM Ca to growing beagles resulted in skeletal changes [3]. Although the test diet fed in this study contained a lower level of Ca (2.83% DM), no observed adverse effects on bone health were observed. The circulating bone formation marker (BAP) and bone resorption marker (CTx) were not significantly different between diet group. Both bone markers declining in both diet groups over time would be expected given that bone turnover is known to decline with age. The onset of decline from baseline in the test group was later than the control group, which could be due to natural variability, environmental factors or higher mineral content of the test diet. BAP levels measured in the current study were higher than those previously reported [53], however this may be due to other factors such as activity level [54,55], which has been reported to be influential. CTx levels measured for both diet groups were within the normal range reported in dogs [56,57]. Given that both bone markers demonstrate a decline over time and values between groups are similar at baseline and 40 weeks, the difference in the onset of decline and slightly higher BAP levels in the test group are unlikely to be clinically meaningful. Furthermore, DXA, ultrasonographic and radiographic examinations showed no evidence of skeletal abnormalities, soft tissue mineralization, urolithiasis, or calculi caused by diet. RSS parameters measured in this study are predictive of mineral crystallization potential leading to the formation of CaOx or struvite uroliths, which are the most prevalent types observed in dogs [58]. Some urinary analytes are known to be influenced by diet in beagles and other breeds [8,10,23]. A cause for hypercalciuria is increased absorption of Ca, which may be exacerbated by a high Ca intake [59]. With no consistent differences between diet group, it is concluded that the test diet fed in this study did not increase the risk of CaOx or struvite uroliths formation in beagles, however, this finding may differ in breeds susceptible to forming CaOx stones or other disease states [8,22,60].

The test diet did not impact protein, energy, or fat digestibilities, which is consistent with findings in Labrador retriever [27]. Furthermore, Labrador retrievers showed no effect of a high Ca diet on lipid metabolites over a 4 h post-prandial time course [61], which is in contrast to findings where Ca was combined with flaxseed mucilage [62] or in other species [63,64,65] where high Ca intake can reduce fat digestibility due to the formation of insoluble fatty acid soaps. Reduced fat absorption could also impact BW and body composition; however, no difference in BW or diet intake were found between diet group. The DM digestibility of the test diet was lower than that of the control diet, which is consistent with findings in Labrador retriever [27] and most likely driven by the difference in dietary ash content (Table 1). DM digestibility of the test diet in the current study was notably higher than that of the Labrador retriever study [27] at week 8; however, was closer at all other time points. This difference may be coincidental or could be linked to Ca and P retention during the early weeks of higher mineral intake in beagles, as indicated by the positive Ca and P balance. High dietary Ca levels has also been reported to decrease zinc absorption in humans [66]. Although not measured in this study, this response was not observed in Labrador retriever and therefore may differ among species [27].

The findings of this study also provide insights into the safety of MCP as an inorganic source of P. Organic phosphates originate from the pork meal and cereals in the diet, whereas other sources of inorganic phosphates include diet additives such as pallatants and carriers of a vitamin premix, which are commonly used in the food manufacture industry [67]. High levels of dietary P has been associated with adverse effects on renal health in several species [28,29,30], however, chronic kidney disease (CKD) has a lower prevalence in dogs than other species such as cats [68,69,70]. A study that demonstrated a link between P and protein intake with CKD in cats, found no such link in dogs [71]. Plasma creatinine, UACR, and specific gravity were not significantly different between diet groups at any point during the current study, and ultrasound examinations found no changes in kidney structure or any evidence of stone formation in any dog at any time point during the course of the study. GFR was not significantly different between diet groups at any time point, however, it declined in both diet groups over the course of the study, significantly different from the baseline at 24 and 40 weeks in the test group. This decline in GFR in both diet groups has been previously observed in dog and cat studies that have run over similar seasons [27,70], therefore this decline may be due to seasonal variation and possibly hydration status, which has been previously reported [72,73,74]. Therefore, the data indicate that P levels of 4.25 g/4184 kJ (1000 kcal) in the test diet, predominantly supplied by MCP, does not result in any observed adverse health effects when fed to adult beagles over a period of 40 weeks. Adult Labrador retrievers also showed no response in these renal health measures when consuming a similar diet for the same length of time [27]. It should be noted that the diets fed in this study do not represent all diets available in the market that have different sources of raw materials and Ca:P ratios, which can alter Ca and P absorption. Although the implications of high dietary P is not fully researched and understood, it has been reported that beagles who were orally administered 0.8 g/kg BW dipotassium phosphate (K_2_ HPO_4_) developed renal damage with accompanying clinical symptoms [75], which underlines source as well as dietary level as additional important factors. Differences in P digestibility and post-prandial serum P and PTH have been demonstrated in adult beagle dogs in response to dietary P derived from different sources [50,76], as has also been found in adult cats [29,49,77]. Whilst these studies showing post-prandial responses provide valuable insights of differing bioavailability and absorption properties from different P sources, it does not provide conclusive evidence of possible health consequences or any protective mechanisms that species and breeds have to eliminate surplus P.

## 5. Conclusions

This study provides evidence that healthy, medium sized breed, adult beagle dogs fed a diet containing 7.19 g Ca/4184 kJ (1000 kcal) balanced with 4.25 g P/4184 kJ (1000 kcal), mainly from inorganic sources, for 40 weeks did not result in observed adverse health. This is in agreement with findings from a previous study conducted in adult Labrador retrievers [27], despite a higher Ca and P exposure (per kg BW^0.75^) in beagles.

## Figures and Tables

**Table 1:** Nutrient composition of the control and test diet. Diet analysis performed at the Mars Petcare Europe Central Laboratory. The analysis used three samples (each sample analyzed in triplicate) from different parts of a 2 kg batch of diet. The values presented in this table are averages and are within conformance standards.

-	Control	Test
Dry matter (g/100 g)	89.76	90.10
Moisture (g/100 g)	10.24	9.90
Protein (g/100 g)	30.53	25.40
Fat (g/100 g)	12.22	12.20
Ash (g/100 g)	4.73	10.30
Total dietary fiber (g/100 g)	6.10	5.41
Calcium (g/4184 kJ (1000 kcal))	1.44	7.19
Phosphorus (g/4184 kJ (1000 kcal))	1.05	4.25
Calcium: Phosphorus ratio	1.38	1.69
Sodium (g/4184 kJ (1000 kcal))	1.12	1.16
Chloride (g/4184 kJ (1000 kcal))	2.02	2.11
Potassium (g/4184 kJ (1000 kcal))	2.28	2.56
Magnesium (mg/4184 kJ (1000 kcal))	184	338
Iron (mg/4184 kJ (1000 kcal))	25.70	55.23
Copper (mg/4184 kJ (1000 kcal))	1.72	2.82
Manganese (mg/4184 kJ (1000 kcal))	19.75	16.06
Zinc (mg/4184 kJ (1000 kcal))	43.56	55.51
Iodine (mg/4184 kJ (1000 kcal))	1.10	1.18
Selenium (µg/4184 kJ (1000 kcal))	115.49 ^†^	121.16
Vitamin A (µg/4184 kJ (µg/1000 kcal))	1441	1454
Vitamin D3 (µg/4184 kJ (µg/1000 kcal))	4.24	4.40
Vitamin E (mg/4184 kJ (mg/1000 kcal))	70	108
Thiamin (mg/4184 kJ (1000 kcal))	2.26	1.86
Riboflavin (mg/4184 kJ (1000 kcal))	1.44	1.04
Pantothenic acid (mg/4184 kJ (1000 kcal))	6.88	7.86
Niacin/nicotinic acid (mg/4184 kJ (1000 kcal))	13.12	13.27
Pyridoxine (mg/4184 kJ (1000 kcal))	2.26	2.48
Folic acid (µg/4184 kJ (1000 kcal))	354	405
Biotin (µg/4184 kJ (1000 kcal))	276	310
Cyanocobalamin (µg/4184 kJ (1000 kcal))	15	14
Choline (g/4184 kJ (1000 kcal))	607	648
Base excess (mEq/kg) ^‡^	96	443
PME § (kcal/100 g)	424.47	393.90

PME, predicted metabolizable energy. ^†^ Analysis performed at Eurofins Ltd. ^‡^ Base excess calculated from methionine and cysteine on a per kg food basis [33]. § Calculated by proximate analysis to PME kcal/100 g [34].

**Table 2:** Bodyweight, energy, calcium, and phosphorus intake values given as means and 95% confidence intervals (CI) for the control (*n =* 8) and test (*n =* 10) diet dog groups at baseline and at eight-week intervals. *p*-Values represent the comparison between diet groups at the sample collection time point.

-	Weeks	Control		Test		*p*
		Mean	95% Cl	Mean	95% Cl	
Bodyweight (kg)	Baseline	13.14	11.07, 15.20	14.00	12.15, 15.85	0.997
	8	13.25	11.19, 15.31	13.99	12.14, 15.84	0.999
	16	13.41	11.35, 15.48	13.75	11.90, 15.60	1.000
	24	13.53	11.46, 15.59	14.05	12.20, 15.90	1.000
	32	13.64	11.57, 15.70	13.80	11.95, 15.65	1.000
	40	13.14	11.07, 15.20	13.78	11.93, 15.63	1.000
Energy intake (kJ/kg ^0.75^)	Baseline	628	532, 724	663	577, 748	0.998
	8	608	512, 704	665	579, 751	0.924
	16	592	496, 688	627	542, 713	0.998
	24	532	436, 628	593	508, 679	0.888
	32	515 *	419, 611	539 *	453, 625	1.000
	40	453 *	358, 549	530 *	444, 616	0.680
Calcium intake (mg/kg ^0.75^) ^†^	Baseline	216	183, 249	228	199, 258	-
	8	209	176, 242	1143	995, 1290	-
	16	204	171, 237	1078	931, 1225	-
	24	183	150, 216	1020	872, 1167	-
	32	177	144, 210	927	779, 1074	-
	40	156	123, 189	911	763, 1058	-
Phosphorus intake (mg/kg ^0.75^) ^†^	Baseline	158	134, 182	166	145, 188	-
	8	153	128, 177	675	588, 762	-
	16	149	125, 173	637	550, 724	-
	24	134	110, 158	603	516, 690	-
	32	129	105, 153	548	461, 635	-
	40	114	90, 138	538	451, 625	

* A significant difference from baseline within diet group (*p* < 0.05). ^†^ Calculated from energy intake (kJ/kg ^0.75^).

**Table 3:** Calcium, phosphorous, and related regulatory hormones and metabolite values measured in blood given as the means and 95% confidence intervals (CI) for the control (*n* = 8) and test (*n* = 10) diet dog groups at the baseline and at eight-week intervals. P values represent the comparison between diet groups at the sample collection time point.

-	Weeks	Control		Test		*p*
		Mean	95% Cl	Mean	95% Cl	
Plasma calcium (mmol/L)	Baseline	2.48	2.42, 2.54	2.40	2.35, 2.45	0.098
	8	2.48	2.42, 2.54	2.44	2.38, 2.49	0.757
	16	2.50	2.44, 2.56	2.44	2.39, 2.49	0.292
	24	2.53 *	2.47, 2.59	2.46 *	2.41, 2.52	0.245
	32	2.54 *	2.48, 2.61	2.49 *	2.44, 2.54	0.505
	40	2.54 *	2.48, 2.60	2.47 *	2.42, 2.53	0.236
Plasma phosphorus (mmol/L)	Baseline	1.24	1.11, 1.39	1.30	1.18, 1.44	0.992
	8	1.21	1.08, 1.36	1.20	1.08, 1.33	1.000
	16	1.29	1.15, 1.45	1.19	1.08, 1.32	0.817
	24	1.22	1.08, 1.36	1.20	1.08, 1.33	1.000
	32	1.26	1.12, 1.41	1.14 *	1.03, 1.26	0.558
	40	1.17	1.04, 1.31	1.15 *	1.04, 1.27	1.000
Ionized calcium (mmol/L)	Baseline	1.29	1.25, 1.33	1.28	1.24, 1.32	0.999
	8	1.18 *	1.14, 1.22	1.29	1.26, 1.33	<0.001 **
	16	1.30	1.26, 1.34	1.31	1.27, 1.34	1.000
	24	1.30	1.26, 1.34	1.32	1.28, 1.36	0.989
	32	1.31	1.26, 1.35	1.30	1.27, 1.34	1.000
	40	1.28	1.24, 1.32	1.29	1.25, 1.32	1.000
Parathyroid hormone (pg/mL)	Baseline	18.27	11.72, 28.47	12.18	8.19, 18.11	0.488
	8	12.38	7.94, 19.29	11.06	7.44, 16.45	1.000
	16	12.32	7.90, 19.19	14.13	9.50, 21.01	1.000
	24	10.33	6.63, 16.09	9.99	6.72, 14.86	1.000
	32	10.58	6.79, 16.49	9.61	6.46, 14.29	1.000
	40	15.26	9.79, 23.78	13.72	9.23, 20.41	1.000
Fibroblast growth factor 23 (pg/mL)	Baseline	336	253, 447	411	319, 530	0.808
	8	351	264, 466	392	304, 505	0.996
	16	311	234, 414	384	298, 496	0.759
	24	293	220, 390	348	270, 449	0.912
	32	323	243, 429	339	263, 438	1.000
	40	178 *	134, 237	209 *	162, 269	0.952
Total calcidiol (25OHD) (nmol/L)	Baseline	70.30	56.97, 86.75	79.10	65.54, 95.47	0.956
	8	73.46	59.53, 90.65	82.98	68.76, 100.15	0.946
	16	69.37	56.21, 85.60	79.06	65.51, 95.42	0.918
	24	75.28	61.01, 92.89	80.81	66.96, 97.53	0.999
	32	82.49 *	66.85, 101.80	86.89	71.99, 104.87	1.000
	40	78.52	63.63, 96.89	86.44	71.63, 104.33	0.990
Calcitriol (1,25(OH)_2_D) (pmol/L)	Baseline	25.10	18.25, 34.53	25.43	19.25, 33.60	1.000
	8	17.49 *	12.55, 24.38	30.40	23.01, 40.17	0.008 **
	16	16.80 *	12.05, 23.41	27.61	20.90, 36.48	0.027 **
	24	21.01	15.23, 29.00	26.72	20.22, 35.31	0.757
	32	17.96 *	13.02, 24.79	23.38	17.61, 31.05	0.660
	40	17.80 *	12.89, 24.56	25.17	19.05, 33.25	0.284
24,25-dihydroxycholecalciferol (24,25(OH)_2_D_3_) (nmol/L)	Baseline	31.12	23.08, 41.98	35.05	26.82, 45.80	0.997
	8	30.84	22.87, 41.59	33.76	25.83, 44.11	1.000
	16	25.56 *	18.95, 34.47	31.23	23.90, 40.81	0.884
	24	23.21 *	17.21, 31.30	26.88 *	20.57, 35.13	0.984
	32	23.48 *	17.41, 31.66	24.22 *	18.54, 31.65	1.000
	40	23.23 *	17.22, 31.33	25.65 *	19.63, 33.51	0.999
24,25-dihydroxyergocalciferol (24,25(OH)D_2_) (nmol/L)	Baseline	9.19	6.33, 13.33	7.06	5.06, 9.85	0.857
	8	9.27	6.39, 13.45	7.89	5.66, 11.01	0.995
	16	7.57 *	5.21, 10.98	6.74	4.83, 9.40	1.000
	24	6.77 *	4.67, 9.83	5.97 *	4.28, 8.32	0.999
	32	6.25 *	4.31, 9.07	5.72 *	4.10, 7.98	1.000
	40	6.79 *	4.68, 9.85	5.69 *	4.08, 7.94	0.989
Total dihydroxycalcidiol (24,25(OH)_2_D) (nmol/L)	Baseline	40.83	31.33, 53.21	42.72	33.71, 54.14	1.000
	8	40.69	31.22, 53.03	42.53	33.56, 53.90	1.000
	16	33.58 *	25.77, 43.77	38.57	30.44, 48.88	0.974
	24	30.51 *	23.41, 39.77	33.45 *	26.39, 42.39	0.999
	32	30.11 *	23.10, 39.24	30.60 *	24.14, 38.77	1.000
	40	30.33 *	23.27, 39.53	31.95 *	25.21, 40.48	1.000
Calcidiol (25OHD_3_) (nmol/L)	Baseline	68.97	55.79, 85.26	77.59	64.19, 93.79	0.959
	8	72.41	58.58, 89.52	81.86	67.72, 98.95	0.947
	16	68.24	55.20, 84.36	78.10	64.60, 94.40	0.907
	24	73.12	59.15, 90.39	79.40	65.68, 95.98	0.997
	32	78.99	63.90, 97.65	83.44	69.03, 100.87	1.000
	40	73.25	59.26, 90.56	83.73	69.27, 101.22	0.912
Ergocalcidiol (25OHD_2_) (nmol/L)	Baseline	1.26	0.79, 1.99	1.47	0.97, 2.22	0.999
	8	1.03	0.65, 1.64	1.09	0.72, 1.64	1.000
	16	1.09	0.69, 1.72	0.92	0.61, 1.40	0.998
	24	1.85	1.17, 2.93	1.27	0.84, 1.92	0.649
	32	2.88 *	1.81, 4.56	2.65 *	1.75, 4.00	1.000
	40	4.34 *	2.74, 6.89	2.51 *	1.66, 3.80	0.165
Total calcidiol: total dihydroxycalcidiol ratio (25OHD:24,25(OH)_2_D)	Baseline	1.72	1.49, 1.98	1.85	1.63, 2.10	0.970
	8	1.81	1.57, 2.08	1.95	1.72, 2.21	0.954
	16	2.07 *	1.79, 2.38	2.05	1.81, 2.33	1.000
	24	2.47 *	2.14, 2.84	2.42 *	2.13, 2.74	1.000
	32	2.74 *	2.38, 3.16	2.84 *	2.50, 3.22	1.000
	40	2.59 *	2.25, 2.98	2.71 *	2.38, 3.07	0.999

* A significant difference from baseline within diet group (*p* < 0.05). ** A significant difference from baseline between diet groups (*p* < 0.05).

**Table 4:** Renal and urinary tract health measure values given as means and 95% confidence intervals (CI) for the control (*n* = 8) and test (*n =* 10) diet dog groups at baseline and at eight-week intervals. *p*-Values represent comparison between diet groups at the sample collection time point.

-	Weeks	Control		Test		*p*
		Mean	95% Cl	Mean	95% Cl	
Plasma creatinine (µmol/L)	Baseline	78.67	71.35, 86.76	85.43	78.27, 93.23	0.674
	8	78.00	70.73, 86.01	85.46	78.30, 93.27	0.541 **
	16	81.15	73.59, 89.49	81.13 *	74.34, 88.55	1.000
	24	87.62 *	79.45, 96.62	91.99 *	84.29, 100.40	0.982
	32	88.25 *	80.03, 97.31	91.68 *	84.01, 100.06	0.997
	40	89.73 *	81.37, 98.94	94.69 *	86.76, 103.35	0.964
Plasma urea (mmol/L)	Baseline	5.20	4.52, 5.98	6.41	5.66, 7.27	0.028
	8	5.54	4.81, 6.37	5.14 *	4.54, 5.83	0.965 **
	16	6.05 *	5.26, 6.97	5.19 *	4.57, 5.88	0.253 **
	24	5.69	4.94, 6.55	5.54 *	4.89, 6.28	1.000 **
	32	6.16 *	5.35, 7.08	5.26 *	4.64, 5.96	0.235 **
	40	5.45	4.74, 6.27	5.26 *	4.64, 5.96	1.000 **
Urine specific gravity	Baseline	1.034	1.034, 1.035	1.034	1.034, 1.035	0.841
	8	1.034	1.034, 1.035	1.034	1.034, 1.035	1.000
	16	1.035	1.034, 1.035	1.035	1.034, 1.035	1.000
	24	1.034	1.034, 1.035	1.035	1.034, 1.035	0.794
	32	1.035 *	1.034, 1.035	1.035	1.034, 1.035	1.000
	40	1.034	1.034, 1.035	1.035	1.034, 1.035	0.429
Urine RSS struvite	Baseline	0.26	0.07, 0.93	1.41	0.46, 4.35	0.075
	8	0.75	0.21, 2.66	0.05 *	0.02, 0.16	<0.001 **
	16	0.70	0.20, 2.48	0.31 *	0.10, 0.97	0.888 **
	24	1.01	0.28, 3.55	0.41	0.13, 1.28	0.811 **
	32	1.10	0.31, 3.89	0.47	0.15, 1.45	0.846 **
	40	2.42 *	0.68, 8.53	0.75	0.24, 2.32	0.477 **
Urine RSS calcium oxalate	Baseline	2.18	1.31, 3.65	5.87	3.71, 9.30	0.001
	8	3.39	2.02, 5.66	3.07 *	1.94, 4.86	1.000 **
	16	3.50	2.09, 5.85	5.70	3.60, 9.02	0.452
	24	3.84	2.30, 6.42	4.83	3.05, 7.65	0.989
	32	2.68	1.60, 4.47	4.76	3.01, 7.54	0.228
	40	3.25	1.94, 5.44	5.11	3.22, 8.09	0.558
Urine albumin: creatinine ratio (mg/g)	Baseline	12.16	4.47, 33.07	5.50	2.25, 13.45	0.738
	8	8.81	3.24, 23.96	8.46	3.46, 20.71	1.000
	16	6.49 *	2.38, 17.64	13.06 *	5.34, 31.97	0.851 **
	24	6.29 *	2.31, 17.10	8.67	3.54, 21.23	0.999 **
	32	8.44	3.10, 22.96	7.73	3.16, 18.92	1.000
	40	5.56 *	2.04, 15.11	8.27	3.38, 20.25	0.997 **
Weight adjusted clearance (ml/min/kg)	Baseline	3.04	2.40, 3.68	3.03	2.46, 3.60	1.000
	8	3.55	2.91, 4.19	2.90	2.32, 3.47	0.335
	16	2.93	2.29, 3.57	2.82	2.25, 3.39	1.000
	24	2.68	2.04, 3.32	2.35 *	1.78, 2.92	0.971
	32	2.53	1.89, 3.17	2.39	1.82, 2.96	1.000
	40	2.40	1.76, 3.04	2.29 *	1.71, 2.86	1.000

RSS, relative super saturation. * A significant difference from baseline within diet group (*p* < 0.05). ** A significant difference from baseline between diet groups (*p* < 0.05).

**Table 5:** Bone health measure values given as means and 95% confidence intervals (CI) for the control (*n =* 8) and test (*n =* 10) diet dog groups at baseline and at eight-week intervals. P values represent the comparison between diet groups at the sample collection time point.

-	Weeks	Control		Test		*p*
		Mean	95% Cl	Mean	95% Cl	
DXA: Bone mineral content (g)	Baseline	393	331, 467	422	362, 492	0.961
	24	404 *	340, 479	429	368, 501	0.979
	40	403	340, 478	429	368, 500	0.978
DXA: Bone mineral density (g/cm^2^)	Baseline	0.820	0.787, 0.855	0.826	0.796, 0.857	0.999
	24	0.822	0.788, 0.856	0.829	0.799, 0.860	0.997
	40	0.819	0.785, 0.853	0.831	0.801, 0.862	0.972
Bone alkaline phosphatase (U/L)	Baseline	14.56	10.69, 19.85	14.90	11.30, 19.66	1.000
	8	11.49	8.43, 15.66	16.56	12.56, 21.84	0.179
	16	10.17 *	7.46, 13.85	15.63	11.85, 20.62	0.058
	24	10.54 *	7.74, 14.37	14.09	10.68, 18.58	0.477
	32	9.33 *	6.85, 12.71	11.00 *	8.34, 14.51	0.962
	40	9.59 *	7.04, 13.06	10.99 *	8.34, 14.50	0.990
Serum cross-laps (ng/mL)	Baseline	0.40	0.22, 0.71	0.47	0.28, 0.79	1.000
	8	0.27 *	0.15, 0.49	0.43	0.26, 0.72	0.730
	16	0.27 *	0.15, 0.48	0.34 *	0.20, 0.57	0.997
	24	0.28	0.16, 0.50	0.32 *	0.19, 0.54	1.000
	32	0.25 *	0.14, 0.44	0.33 *	0.20, 0.56	0.976
	40	0.25 *	0.14, 0.44	0.26 *	0.15, 0.43	1.000

DXA, dual energy x-ray absorptiometry. * A significant difference from the baseline within diet group *(p* < 0.05).

## Data Availability

All additional data are provided as Supplementary Materials to this article.

## Supplementary Materials

The following are available online at https://www.mdpi.com/article/10.3390/ani11061799/s1, Table S1: Additional nutrient composition of the control and test diet. Table S2: Additional plasma biochemistry, Table S3: Hematology, Table S4: Diet digestibility, mineral excretion, and balance.

## References

[1] De Brito Galvao J.F., Nagode L.A., Schenck P.A., Chew D.J. (2013). Calcitriol, calcidiol, parathyroid hormone, and fibroblast growth factor-23 interactions in chronic kidney disease. J. Vet. Emerg. Crit. Care.

[2] Jeon U.S. (2008). Kidney and calcium homeostasis. Electrolyte Blood Press.

[3] Dobenecker B., Kasbeitzer N., Flinspach S., Köstlin R., Matis U., Kienzle E. (2006). Calcium-excess causes subclinical changes of bone growth in Beagles but not in Foxhound-crossbred dogs, as measured in X-rays. J. Anim. Physiol. Anim. Nutr..

[4] Nap R.C., Hazewinkel H.A. (1994). Growth and skeletal development in the dog in relation to nutrition; a review. Vet. Q..

[5] Schoenmakers I., Hazewinkel H.A., Voorhout G., Carlson C.S., Richardson D. (2000). Effects of diets with different calcium and phosphorus contents on the skeletal development and blood chemistry of growing great danes. Vet. Rec..

[6] Goedegebuure S.A., Hazewinkel H.A. (1986). Morphological findings in young dogs chronically fed a diet containing excess calcium. Vet. Pathol..

[7] Cortadellas O., Fernández del Palacio M.J., Talavera J., Bayón A. (2010). Calcium and Phosphorus Homeostasis in Dogs with Spontaneous Chronic Kidney Disease at Different Stages of Severity. J. Vet. Intern. Med..

[8] Lulich J.P., Osborne C.A., Nagode L.A., Polzin D.J., Parke M.L. (1991). Evaluation of urine and serum metabolites in miniature schnauzers with calcium oxalate urolithiasis. Am. J. Vet. Res..

[9] Dobenecker B. (2011). Factors that modify the effect of excess calcium on skeletal development in puppies. Br. J. Nutr..

[10] Kiefer-Hecker B., Kienzle E., Dobenecker B. (2018). Effects of low phosphorus supply on the availability of calcium and phosphorus, and musculoskeletal development of growing dogs of two different breeds. J. Anim. Physiol. Anim. Nutr..

[11] Laflamme G.H., Jowsey J. (1972). Bone and soft tissue changes with oral phosphate supplements. J. Clin. Investig..

[12] Cook S.D., Skinner H.B., Haddad R.J. (1983). A quantitative histologic study of osteoporosis produced by nutritional secondary hyperparathyroidism in dogs. Clin. Orthop. Relat. Res..

[13] Saville P.D., Krook L. (1969). Gravimetric and isotopic studies in nutritional hyperparathyroidism in beagles. Clin. Orthop. Relat. Res..

[14] Cloutier M., Gascon-Barré M., D’Amour P. (1992). Chronic adaptation of dog parathyroid function to a low-calcium-high-Sodium-Vitamin D-deficient diet. J. Bone Miner. Res..

[15] Stockman J., Villaverde C., Corbee R.J. (2021). Calcium, Phosphorus, and Vitamin D in Dogs and Cats: Beyond the Bones. Vet. Clin. N. Am. Small Anim. Pract..

[16] Mack J.K., Alexander L.G., Morris P.J., Dobenecker B., Kienzle E. (2015). Demonstration of uniformity of calcium absorption in adult dogs and cats. J. Anim. Physiol. Anim. Nutr..

[17] National Research Council (2006). Nutrient Requirements of Dogs and Cats.

[18] Schmitt S., Mack J., Kienzle E., Alexander L.G., Morris P.J., Colyer A., Dobenecker B. (2018). Faecal calcium excretion does not decrease during long-term feeding of a low-calcium diet in adult dogs. J. Anim. Physiol. Anim. Nutr..

[19] Wilkens M.R., Richter J., Fraser D.R., Liesegang A., Breves G., Schroder B. (2012). In contrast to sheep, goats adapt to dietary calcium restriction by increasing intestinal absorption of calcium. Comp. Biochem. Physiol. A Mol. Integr. Physiol..

[20] Liesegang A., Sassi M., Risteli J., Kraenzlin M., Riond J., Wanner M. (1999). Influence of low calcium, high calcium and energy-rich diets on bone markers in ovariectomized Beagle dogs. J. Anim. Physiol. Anim. Nutr..

[21] Stephens L.C., Norrdin R.W., Benjamin S.A. (1985). Effects of calcium supplementation and sunlight exposure on growing beagle dogs. Am. J. Vet. Res..

[22] Furrow E., Patterson E.E., Armstrong P.J., Osborne C.A., Lulich J.P. (2015). Fasting urinary calcium-to-creatinine and oxalate-to-creatinine ratios in dogs with calcium oxalate urolithiasis and breed-matched controls. J. Vet. Intern. Med..

[23] Lulich J.P., Osborne C.A., Polzin D.J., Johnston S.D., Parker M.L. (1991). Urine metabolite values in fed and nonfed clinically normal beagles. Am. J. Vet. Res..

[24] Slater M.R., Scarlett J.M., Donoghue S., Kaderly R.E., Bonnett B.N., Cockshutt J., Erb H.N. (1992). Diet and exercise as potential risk factors for osteochondritis dissecans in dogs. Am. J. Vet. Res..

[25] Association of American Feed Control Officials (AAFCO) (2018). 2018 Official Publication.

[26] European Pet Food Industry Federation (FEDIAF) (2019). Nutritional Guidelines for Complete and Complementary Pet Food for Cats and Dogs.

[27] Stockman J., Watson P., Gilham M., Allaway D., Atwal J., Haydock R., Colyer A., Renfrew H., Morris P.J. (2017). Adult dogs are capable of regulating calcium balance, with no adverse effects on health, when fed a high-calcium diet. Br. J. Nutr..

[28] Matsuzaki H., Kikuchi T., Kajita Y., Masuyama R., Uehara M., Goto S., Suzuki K. (1999). Comparison of various phosphate salts as the dietary phosphorus source on nephrocalcinosis and kidney function in rats. J. Nutr. Sci. Vitam..

[29] Pastoor F.J., Van’t Klooster A.T., Mathot J.N., Beynen A.C. (1995). Increasing phosphorus intake reduces urinary concentrations of magnesium and calcium in adult ovariectomized cats fed purified diets. J. Nutr..

[30] Nadkarni G.N., Uribarri J. (2014). Phosphorus and the Kidney: What Is Known and What Is Needed. Adv. Nutr..

[31] German A.J., Holden S.L., Moxham G.L., Holmes K.L., Hackett R.M., Rawlings J.M. (2006). A simple, reliable tool for owners to assess the body condition of their dog or cat. J. Nutr..

[32] Association of American Feed Control Officials (AAFCO) (2013). 2013 Official Publication.

[33] Kienzle E., Wilms-Eilers S. (1994). Struvite Diet in Cats: Effect of Ammonium Chloride and Carbonates on Acid Base Balance of Cats. J. Nutr..

[34] Laflamme D.P. (2001). Determining metabolizable energy content in commercial pet foods. J. Anim. Physiol. Anim. Nutr..

[35] (2009). European Union, Regulation (EC) N °152/of the commission of 27 January 2009. Off. J. Eur. Union.

[36] Robertson W.G., Jones J.S., Heaton M.A., Stevenson A.E., Markwell P.J. (2002). Predicting the Crystallization Potential of Urine from Cats and Dogs with Respect to Calcium Oxalate and Magnesium Ammonium Phosphate (Struvite). J. Nutr..

[37] Estepa J.C., Lopez I., Felsenfeld A.J., Gao P., Cantor T., Rodríguez M., Aguilera-Tejero E. (2003). Dynamics of secretion and metabolism of PTH during hypo- and hypercalcaemia in the dog as determined by the ‘intact’ and ‘whole’ PTH assays. Nephrol. Dial. Transplant..

[38] Mooney C.T., Shiel R.E., Fawcett K., Matthews E., Gunn E. (2019). A comparison of canine whole and intact parathyroid hormone concentrations as measured by different assays. J. Small Anim. Pract..

[39] Mooney C.T., Shiel R.E., Sekiya M., Dunning M., Gunn E. (2020). A Preliminary Study of the Effect of Hyperadrenocorticism on Calcium and Phosphate Concentrations, Parathyroid Hormone and Markers of Bone Turnover in Dogs. Front. Vet. Sci..

[40] Harjes L.M., Parker V.J., Dembek K., Young G.S., Giovaninni L.H., Kogika M.M., Chew D.J., Toribio R.E. (2017). Fibroblast Growth Factor-23 Concentration in Dogs with Chronic Kidney Disease. J. Vet. Intern. Med..

[41] Tang J.C.Y., Nicholls H., Piec I., Washbourne C.J., Dutton J.J., Jackson S., Greeves J., Fraser W.D. (2017). Reference intervals for serum 24,25-Dihydroxyvitamin D and the ratio with 25-Hydroxyvitamin established using a newly developed LC-MS/MS method. J. Nutr. Biochem..

[42] Bexfield N.H., Heiene R., Gerritsen R.J., Risoen U., Eliassen K.A., Herrtage M.E., Michell A.R. (2008). Glomerular filtration rate estimated by 3-sample plasma clearance of iohexol in 118 healthy dogs. J. Vet. Intern. Med..

[43] Munday H.S., Booles D., Anderson P., Poore D.W., Earle K.E. (1994). The Repeatability of Body Composition Measurements in Dogs and Cats using Dual Energy X-Ray Absorptiometry. J. Nutr..

[44] Speakman J., Booles D., Butterwick R. (2001). Validation of dual energy X-ray absorptiometry (DXA) by comparison with chemical analysis of dogs and cats. Int. J. Obes..

[45] R Core Team (2018). R: A Language and Environment for Statistical Computing.

[46] Bates D., Mächler M., Bolker B., Walker S. (2015). Fitting Linear Mixed-Effects Models Using lme4. arXiv.

[47] Hothorn T., Bretz F., Westfall P. (2008). Simultaneous inference in general parametric models. Biom. J..

[48] Bronner F. (2003). Mechanisms of intestinal calcium absorption. J. Cell. Biochem..

[49] Coltherd J.C., Staunton R., Colyer A., Thomas G., Gilham M., Logan D.W., Butterwick R., Watson P. (2019). Not all forms of dietary phosphorus are equal: An evaluation of postprandial phosphorus concentrations in the plasma of the cat. Br. J. Nutr..

[50] Siedler S., Dobenecker B. The source of phosphorus influences serum PTH, apparent digestibility and blood levels of calcium and phosphorus in dogs fed high phosphorus diets with balanced Ca/P ratio. Proceedings of the Waltham International Nutritional Sciences Symposium.

[51] Liesegang A., Reutter R., Sassi M.L., Risteli J., Kraenzlin M., Riond J.L., Wanner M. (1999). Diurnal variation in concentrations of various markers of bone metabolism in dogs. Am. J. Vet. Res..

[52] López I., Aguilera-Tejero E., Estepa J.C., Bas S., Mayer-Valor R., Jiménez A., Rodríguez M. (2005). Diurnal variations in the plasma concentration of parathyroid hormone in dogs. Vet. Rec..

[53] Allen L.C., Allen M.J., Breur G.J., Hoffmann W.E., Richardson D.C. (2000). A comparison of two techniques for the determination of serum bone-specific alkaline phosphatase activity in dogs. Res. Vet. Sci..

[54] Menkes A., Mazel S., Redmond R.A., Koffler K., Libanati C.R., Gundberg C.M., Zizic T.M., Hagberg J.M., Pratley R.E., Hurley B.F. (1993). Strength training increases regional bone mineral density and bone remodeling in middle-aged and older men. J. Appl. Physiol..

[55] Vrbanac Z., Brkljaca Bottegaro N., Skrlin B., Bojanic K., Kusec V., Stanin D., Belic M. (2020). The Effect of a Moderate Exercise Program on Serum Markers of Bone Metabolism in Dogs. Animals.

[56] Belic M., Kusec V., Svetina A., Grizelj J., Robic M., Vrbanac Z., Benic M., Turk R. (2012). The influence of sex on biochemical markers of bone turnover in dogs. Res. Vet. Sci..

[57] Lucas P.W., Fan T.M., Garrett L.D., Griffon D.J., Wypij J.M. (2008). A comparison of five different bone resorption markers in osteosarcoma-bearing dogs, normal dogs, and dogs with orthopedic diseases. J. Vet. Intern. Med..

[58] Osborne C.A., Lulich J.P., Polzin D.J., Sanderson S.L., Koehler L.A., Ulrich L.K., Bird K.A., Swanson L.L., Pederson L.A., Sudo S.Z. (1999). Analysis of 77,000 canine uroliths: Perspectives from the Minnesota Urolith Center. Vet. Clin. Small Anim. Pract..

[59] Luskin A.C., Lulich J.P., Gresch S.C., Furrow E. (2019). Bone resorption in dogs with calcium oxalate urolithiasis and idiopathic hypercalciuria. Res. Vet. Sci..

[60] Groth E.M., Lulich J.P., Chew D.J., Parker V.J., Furrow E. (2019). Vitamin D metabolism in dogs with and without hypercalciuric calcium oxalate urolithiasis. J. Vet. Intern. Med..

[61] Allaway D., Gilham M., Wagner-Golbs A., Maldonado S.G., Haydock R., Colyer A., Stockman J., Watson P. (2019). Metabolomic profiling to identify effects of dietary calcium reveal the influence of the individual and postprandial dynamics on the canine plasma metabolome. J. Nutr. Sci..

[62] Nybroe S., Astrup A., Bjørnvad C.R. (2016). Dietary supplementation with flaxseed mucilage alone or in combination with calcium in dogs: Effects on apparent digestibility of fat and energy and fecal characteristics. Int. J. Obes..

[63] Jacobsen R., Lorenzen J.K., Toubro S., Krog-Mikkelsen I., Astrup A. (2005). Effect of short-term high dietary calcium intake on 24-h energy expenditure, fat oxidation, and fecal fat excretion. Int. J. Obes..

[64] Papakonstantinou E., Flatt W.P., Huth P.J., Harris R.B. (2003). High dietary calcium reduces body fat content, digestibility of fat, and serum vitamin D in rats. Obes. Res..

[65] Appleton G.V.N., Owen R.W., Williamson R.C.N. (1992). The effect of dietary calcium supplementation on intestinal lipid metabolism. J. Steroid Biochem. Mol. Biol..

[66] Wood R.J., Zheng J.J. (1997). High dietary calcium intakes reduce zinc absorption and balance in humans. Am. J. Clin. Nutr..

[67] Gutierrez O.M. (2013). Sodium- and phosphorus-based food additives: Persistent but surmountable hurdles in the management of nutrition in chronic kidney disease. Adv. Chronic Kidney Dis..

[68] O’Neill D.G., Elliott J., Church D.B., McGreevy P.D., Thomson P.C., Brodbelt D.C. (2013). Chronic kidney disease in dogs in UK veterinary practices: Prevalence, risk factors, and survival. J. Vet. Intern. Med..

[69] Lulich J., Osborne C., O’brien T., Polzin D. (1992). Feline renal failure: Questions, answers, questions. Compend. Contin. Educ. Pract. Vet..

[70] Alexander J., Stockman J., Atwal J., Butterwick R., Colyer A., Elliott D., Gilham M., Morris P., Staunton R., Renfrew H. (2018). Effects of the long-term feeding of diets enriched with inorganic phosphorus on the adult feline kidney and phosphorus metabolism. Br. J. Nutr..

[71] Böswald L.F., Kienzle E., Dobenecker B. (2018). Observation about phosphorus and protein supply in cats and dogs prior to the diagnosis of chronic kidney disease. J. Anim. Physiol. Anim. Nutr..

[72] Tsuda T., Ide M., Iigo M. (1995). Influences of season and of temperature, photoperiod, and subcutaneous melatonin infusion on the glomerular filtration rate of ewes. J. Pineal. Res..

[73] Masugata H., Senda S., Inukai M., Himoto T., Murao K., Hosomi N., Iwado Y., Noma T., Kohno M., Goda F. (2011). Seasonal Variation in Estimated Glomerular Filtration Rate Based on Serum Creatinine Levels in Hypertensive Patients. Tohoku J. Exp. Med..

[74] Von Hendy-Willson V.E., Pressler B.M. (2011). An overview of glomerular filtration rate testing in dogs and cats. Vet. J..

[75] Schneider P., Pappritz G., Muller-Peddinghaus R., Bauer M., Lehmann H., Ueberberg H., Trautwein G. (1980). Potassium hydrogen phosphate induced nephropathy in the dog. I. Pathogenesis of tubular atrophy (author’s transl). Vet. Pathol..

[76] Dobenecker B., Reese S., Herbst S. (2021). Effects of dietary phosphates from organic and inorganic sources on parameters of phosphorus homeostasis in healthy adult dogs. PLoS ONE.

[77] Dobenecker B., Hertel-Böhnke P., Webel A., Kienzle E. (2018). Renal phosphorus excretion in adult healthy cats after the intake of high phosphorus diets with either calcium monophosphate or sodium monophosphate. J. Anim. Physiol. Anim. Nutr..

