# Supplementary material for: No Observed Adverse Effects on Health Were Detected in Adult Beagle Dogs When Fed a High-Calcium Diet for 40 Weeks
Source: Animals (Basel). 2021 Jun 16;11(6):1799. doi: 10.3390/ani11061799 (PMC8234157; doi:10.3390/ani11061799)
Supplement: Supplementary file 1 [file animals-11-01799-s001.zip › Table_S1.pdf]

Table S1. Additional nutrient composition of the control and test diet

Additional nutrient composition of the control and test diet. Diet analysis performed at the Mars Petcare Europe Central Laboratory. The analysis used three samples (each sample analysed in triplicate) from different parts of a 2kg batch of diet. The values presented in this table are averages and are within conformance standards.

	Control	Test
Linoleic acid (g/4184 kJ (1000 kcal))	4.62	5.21
Arachidonic acid (g/4184 kJ (1000 kcal))	0.10	0.11
Total omega-6 fatty acid (g/4184 kJ (1000 kcal))	4.99	5.52
Docosahexaenoic acid and eicosapentaenoic acid (g/4184 kJ (1000 kcal))	0.50	0.68
Alpha linoleic acid (g/4184 kJ (1000 kcal))	0.39	0.45
Total omega-3 fatty acid (g/4184 kJ (1000 kcal))	1.08	1.27
Arginine (g/4184 kJ (1000 kcal))	5.33	4.71
Cystine (g/4184 kJ (1000 kcal))	0.76	0.73
Histidine (g/4184 kJ (1000 kcal))	1.65	1.49
Hydroxyproline (g/4184 kJ (1000 kcal))	4.07	4.40
Isoleucine (g/4184 kJ (1000 kcal))	2.34	2.11
Leucine (g/4184 kJ (1000 kcal))	4.93	4.42
Lysine (g/4184 kJ (1000 kcal))	3.91	3.44
Methionine and cystine (g/4184 kJ (1000 kcal))	3.15	3.04
Methionine (g/4184 kJ (1000 kcal))	2.39	2.31
Glutamine (g/4184 kJ (1000 kcal))	11.31	10.00
Phenylalanine (g/4184 kJ (1000 kcal))	2.76	2.48
Threonine (g/4184 kJ (1000 kcal))	2.62	2.23
Tryptophan (g/4184 kJ (1000 kcal))	0.55 †	0.48
Tyrosine (g/4184 kJ (1000 kcal))	1.81	1.58
Valine (g/4184 kJ (1000 kcal))	3.31	2.73

† Analysis performed at Eurofins Ltd.
