# Supplementary material for: No Observed Adverse Effects on Health Were Detected in Adult Beagle Dogs When Fed a High-Calcium Diet for 40 Weeks
Source: Animals (Basel). 2021 Jun 16;11(6):1799. doi: 10.3390/ani11061799 (PMC8234157; doi:10.3390/ani11061799)
Supplement: Supplementary file 1 [file animals-11-01799-s001.zip › Table_S2.pdf]

Table S2. Additional plasma biochemistry

Additional plasma biochemistry values given as means and 95% confidence intervals (CI) for control (*n* 8) and test (*n* 10) diet dog groups at baseline and at 8 week intervals. P values represent comparison between diet groups at the sample collection time point ($p < 0.05$ is statistically signficant).

	Weeks	Control			Test			*P*
			Mean	95% Cl		Mean	95% Cl	
Albumin (g/L)	Baseline	*n* 8	32.38	31.13, 33.68	*n* 10	30.33	29.28, 31.42	0.013
	8	*n* 8	32.87	31.60, 34.18	*n* 10	30.80	29.74, 31.90	0.013
	16	*n* 8	32.23	30.99, 33.53	*n* 10	30.73	29.67, 31.83	0.171
	24	*n* 8	32.63	31.38, 33.94	*n* 10	30.80	29.74, 31.90	0.044
	32	*n* 8	33.22	31.94, 34.56	*n* 10	31.39*	30.30, 32.51	0.051
	40	*n* 8	32.94	31.67, 34.27	*n* 10	30.42	29.37, 31.51	<0.001
Total protein (g/L)	Baseline	*n* 8	59.19	57.24, 61.21	*n* 10	54.64	53.03, 56.31	<0.001
	8	*n* 8	58.48	56.55, 60.47	*n* 10	55.64	53.99, 57.33	0.038
	16	*n* 8	58.69	56.75, 60.69	*n* 10	55.74	54.09, 57.44	0.027
	24	*n* 8	59.05	57.10, 61.06	*n* 10	56.03	54.37, 57.74	0.022
	32	*n* 8	59.40	57.44, 61.43	*n* 10	57.79*	56.09, 59.56	0.671**
	40	*n* 8	60.31	58.32, 62.37	*n* 10	57.40*	55.71, 59.15	0.041
Alanine aminotransferase (U/L)	Baseline	*n* 8	33.06	20.84, 52.44	*n* 10	31.31	20.72, 47.30	1.000
	8	*n* 8	31.23	19.69, 49.53	*n* 10	28.81	19.07, 43.52	1.000
	16	*n* 8	34.13	21.52, 54.14	*n* 10	28.46	18.84, 43.00	0.998
	24	*n* 8	36.15	22.79, 57.34	*n* 10	27.76	18.38, 41.95	0.960
	32	*n* 8	29.38	18.52, 46.60	*n* 10	27.90	18.47, 42.16	1.000
	40	*n* 8	32.88	20.73, 52.15	*n* 10	28.24	18.69, 42.66	1.000
Alkaline phosphatase (U/L)	Baseline	*n* 8	60.08	42.85, 84.24	*n* 10	71.50	52.85, 96.74	0.977
	8	*n* 8	54.32	38.75, 76.17	*n* 10	72.48	53.58, 98.07	0.654
	16	*n* 8	48.82*	34.82, 68.45	*n* 10	66.98	49.51, 90.62	0.533
	24	*n* 8	45.96*	32.78, 64.45	*n* 10	62.27	46.02, 84.24	0.588
	32	*n* 8	45.00*	32.10, 63.10	*n* 10	63.17	46.69, 85.47	0.437
	40	*n* 8	44.23*	31.55, 62.01	*n* 10	59.73	44.15, 80.81	0.602
Aspartate aminotransferase (U/L)	Baseline	*n* 8	26.87	21.91, 32.95	*n* 10	26.59	22.15, 31.91	1.000
	8	*n* 8	28.64	23.36, 35.12	*n* 10	26.38	21.98, 31.66	0.995
	16	*n* 8	26.39	21.52, 32.36	*n* 10	28.26	23.55, 33.92	0.999
	24	*n* 8	27.49	22.41, 33.71	*n* 10	26.22	21.85, 31.47	1.000
	32	*n* 8	25.70	20.96, 31.52	*n* 10	25.54	21.28, 30.65	1.000
	40	*n* 8	26.41	21.54, 32.39	*n* 10	25.77	21.47, 30.92	1.000
Cholesterol (mmol/L)	Baseline	*n* 8	5.05	4.45, 5.73	*n* 10	4.67	4.17, 5.23	0.926
	8	*n* 8	5.19	4.57, 5.89	*n* 10	5.04*	4.50, 5.64	1.000
	16	*n* 8	5.09	4.49, 5.78	*n* 10	5.02*	4.48, 5.62	1.000
	24	*n* 8	4.91	4.33, 5.57	*n* 10	4.98	4.45, 5.58	1.000
	32	*n* 8	4.92	4.33, 5.58	*n* 10	4.90	4.38, 5.49	1.000
	40	*n* 8	4.96	4.37, 5.63	*n* 10	5.09*	4.54, 5.70	1.000**
Triglycerides (mmol/L)	Baseline	*n* 8	0.45	0.32, 0.63	*n* 10	0.43	0.32, 0.58	1.000
	8	*n* 8	0.50	0.36, 0.70	*n* 10	0.41	0.31, 0.55	0.927
	16	*n* 8	0.57	0.41, 0.80	*n* 10	0.50	0.37, 0.67	0.996
	24	*n* 8	0.58	0.42, 0.81	*n* 10	0.47	0.35, 0.64	0.908
	32	*n* 8	0.60	0.43, 0.84	*n* 10	0.55	0.41, 0.74	1.000
	40	*n* 8	0.57	0.41, 0.80	*n* 10	0.53	0.39, 0.71	1.000
Sodium (mmol/L)	Baseline	*n* 8	145.12	142.44, 147.85	*n* 10	145.07	142.67, 147.51	1.000
	8	*n* 8	146.24	143.54, 148.98	*n* 10	145.77	143.36, 148.22	1.000
	16	*n* 8	144.20	141.54, 146.91	*n* 10	144.45	142.07, 146.88	1.000
	24	*n* 8	144.63	141.97, 147.35	*n* 10	137.35*	135.08, 139.66	<0.001**
	32	*n* 8	143.98	141.33, 146.69	*n* 10	145.15	142.76, 147.59	0.991
	40	*n* 8	145.45	142.77, 148.19	*n* 10	145.82	143.42, 148.27	1.000
Potassium (mmol/L)	Baseline	*n* 8	4.19	3.94, 4.45	*n* 10	4.16	3.94, 4.39	1.000

	8	*n* 8	4.14	3.90, 4.40	*n* 10	4.12	3.90, 4.35	1.000
	16	*n* 8	4.23	3.98, 4.49	*n* 10	4.18	3.96, 4.41	1.000
	24	*n* 8	4.12	3.87, 4.37	*n* 10	3.87*	3.66, 4.08	0.356
	32	*n* 8	4.14	3.90, 4.40	*n* 10	4.08	3.87, 4.31	1.000
	40	*n* 8	4.15	3.91, 4.41	*n* 10	4.09	3.88, 4.32	1.000
Chloride (mmol/L)	Baseline	*n* 8	111.87	109.61, 114.18	*n* 10	111.66	109.64, 113.71	1.000
	8	*n* 8	112.49	110.22, 114.81	*n* 10	113.88	111.82, 115.98	0.917
	16	*n* 8	111.61	109.35, 113.91	*n* 10	112.33	110.30, 114.40	0.999
	24	*n* 8	111.37	109.12, 113.67	*n* 10	106.32*	104.40, 108.28	<0.001**
	32	*n* 8	111.45	109.19, 113.75	*n* 10	113.53	111.48, 115.63	0.496
	40	*n* 8	112.68	110.40, 115.01	*n* 10	113.57	111.52, 115.67	0.996
Glucose (mmol/L)	Baseline	*n* 8	5.76	5.37, 6.17	*n* 10	5.67	5.33, 6.03	1.000
	8	*n* 8	5.66	5.28, 6.06	*n* 10	5.57	5.24, 5.93	1.000
	16	*n* 8	5.73	5.35, 6.14	*n* 10	5.48	5.15, 5.83	0.895
	24	*n* 8	5.77	5.38, 6.18	*n* 10	5.67	5.33, 6.03	1.000
	32	*n* 8	5.72	5.34, 6.13	*n* 10	5.52	5.19, 5.88	0.978
	40	*n* 8	5.66	5.29, 6.07	*n* 10	5.48	5.15, 5.83	0.981

* A significant difference from baseline within diet group ($p < 0.05$)

** A significant difference from baseline between diet groups ($p < 0.05$)
