# Supplementary material for: No Observed Adverse Effects on Health Were Detected in Adult Beagle Dogs When Fed a High-Calcium Diet for 40 Weeks
Source: Animals (Basel). 2021 Jun 16;11(6):1799. doi: 10.3390/ani11061799 (PMC8234157; doi:10.3390/ani11061799)
Supplement: Supplementary file 1 [file animals-11-01799-s001.zip › Table_S3.pdf]

Table S3. Haematology

Additional haematology values given as means and 95% confidence intervals (CI) for control (*n* 8) and test (*n* 10) diet dog groups at baseline and at 8 week intervals. P values represent comparison between diet groups at the sample collection time point ($p < 0.05$ is statistically signficant).

	Weeks	Control			Test			*P*
			Mean	95% Cl		Mean	95% Cl	
Red blood cells (10^12/L)	Baseline	*n* 8	6.12	5.72, 6.52	*n* 10	5.70	5.34, 6.06	0.303
	8	*n* 8	6.10	5.70, 6.50	*n* 10	6.25*	5.89, 6.61	0.997
	16	*n* 8	6.21	5.81, 6.60	*n* 10	6.06	5.70, 6.41	0.997
	24	*n* 8	6.10	5.70, 6.50	*n* 10	6.28*	5.92, 6.64	0.991
	32	*n* 2	6.07	5.38, 6.76	*n* 10	6.01	5.65, 6.37	1.000
	40	*n* 8	6.36	5.96, 6.76	*n* 10	6.16*	5.80, 6.52	0.979
Haemoglobin (g/dL)	Baseline	*n* 8	16.61	15.41, 17.91	*n* 10	15.66	14.64, 16.75	0.708
	8	*n* 8	16.84	15.62, 18.15	*n* 10	16.99*	15.88, 18.17	1.000
	16	*n* 8	16.69	15.48, 17.99	*n* 10	15.62	14.60, 16.70	0.549
	24	*n* 8	16.28	15.10, 17.55	*n* 10	16.23	15.17, 17.36	1.000
	32	*n* 2	16.41	14.36, 18.77	*n* 10	15.74	14.71, 16.83	0.997
	40	*n* 8	17.06	15.82, 18.40	*n* 10	16.33	15.27, 17.47	0.934
Hematocrit (%)	Baseline	*n* 8	47.60	44.53, 50.89	*n* 10	44.07	41.52, 46.77	0.187
	8	*n* 8	47.77	44.69, 51.06	*n* 10	48.37*	45.57, 51.34	1.000
	16	*n* 8	47.94	44.85, 51.25	*n* 10	46.65	43.95, 49.51	0.995
	24	*n* 8	47.34	44.28, 50.60	*n* 10	47.53*	44.77, 50.45	1.000
	32	*n* 2	47.37	42.02, 53.40	*n* 10	46.13	43.46, 48.96	1.000
	40	*n* 8	49.19	46.01, 52.59	*n* 10	46.82	44.11, 49.70	0.766
Mean corpuscular volume (fL)	Baseline	*n* 8	77.98	75.57, 80.40	*n* 10	77.87	75.70, 80.03	1.000
	8	*n* 8	78.48	76.06, 80.89	*n* 10	77.56	75.40, 79.72	0.998
	16	*n* 8	77.39	74.98, 79.81	*n* 10	77.31	75.14, 79.47	1.000
	24	*n* 8	77.66	75.24, 80.07	*n* 10	75.84*	73.68, 78.00	0.805
	32	*n* 2	78.50	75.63, 81.37	*n* 10	76.84	74.68, 79.00	0.930
	40	*n* 8	77.56	75.14, 79.97	*n* 10	76.21*	74.04, 78.37	0.963
Mean corpuscular haemoglobin (pg)	Baseline	*n* 8	27.21	26.21, 28.20	*n* 10	27.69	26.80, 28.58	0.982
	8	*n* 8	27.66	26.66, 28.65	*n* 10	27.26	26.37, 28.15	0.996
	16	*n* 8	26.96	25.96, 27.95	*n* 10	25.91*	25.02, 26.80	0.330**
	24	*n* 8	26.70	25.70, 27.70	*n* 10	25.89*	25.00, 26.78	0.678**
	32	*n* 2	27.22	25.74, 28.70	*n* 10	26.22*	25.33, 27.11	0.736
	40	*n* 8	26.90	25.90, 27.90	*n* 10	26.58*	25.68, 27.47	0.999
Mean corpuscular haemoglobin concentration (g/dL)	Baseline	*n* 8	34.89	34.19, 35.59	*n* 10	35.54	34.91, 36.17	0.475
	8	*n* 8	35.26	34.56, 35.96	*n* 10	35.14	34.51, 35.76	1.000
	16	*n* 8	34.81	34.11, 35.51	*n* 10	33.52*	32.89, 34.14	0.001**
	24	*n* 8	34.39	33.69, 35.09	*n* 10	34.15*	33.52, 34.77	0.999
	32	*n* 2	34.41	33.03, 35.79	*n* 10	34.13*	33.50, 34.75	1.000
	40	*n* 8	34.68	33.97, 35.39	*n* 10	34.87	34.24, 35.50	1.000
Red blood cell distribution width (%)	Baseline	*n* 8	12.34	11.73, 12.95	*n* 10	12.02	11.47, 12.57	0.966
	8	*n* 8	11.94	11.33, 12.55	*n* 10	12.75*	12.20, 13.29	0.088**
	16	*n* 8	12.44	11.83, 13.05	*n* 10	12.77*	12.22, 13.32	0.964
	24	*n* 8	12.56	11.95, 13.17	*n* 10	12.97*	12.42, 13.52	0.854
	32	*n* 2	12.17	11.21, 13.12	*n* 10	12.68*	12.13, 13.23	0.904
	40	*n* 8	12.47	11.86, 13.09	*n* 10	12.90*	12.35, 13.45	0.834
White blood cells (10^9/L)	Baseline	*n* 8	6.41	4.94, 7.87	*n* 10	7.08	5.77, 8.39	0.988
	8	*n* 8	6.55	5.09, 8.01	*n* 10	6.37	5.07, 7.68	1.000
	16	*n* 8	6.10	4.64, 7.56	*n* 10	5.92*	4.62, 7.23	1.000
	24	*n* 8	5.98	4.52, 7.44	*n* 10	6.30	5.00, 7.61	1.000
	32	*n* 2	5.91	3.85, 7.96	*n* 10	6.22	4.92, 7.53	1.000
	40	*n* 8	5.59	4.13, 7.05	*n* 10	5.84*	4.54, 7.15	1.000
Lymphocytes (10^9/L)	Baseline	*n* 8	1.15	0.80, 1.66	*n* 10	1.17	0.84, 1.63	1.000
	8	*n* 8	1.22	0.84, 1.76	*n* 10	1.05	0.76, 1.45	0.995
	16	*n* 8	1.28	0.89, 1.85	*n* 10	1.14	0.82, 1.58	0.999

	24	*n* 8	1.21	0.84, 1.75	*n* 10	1.28	0.92, 1.77	1.000
	32	*n* 2	1.35	0.81, 2.26	*n* 10	1.24	0.89, 1.72	1.000
	40	*n* 8	1.14	0.79, 1.64	*n* 10	1.45	1.04, 2.01	0.878
Monocytes	Baseline	*n* 8	0.64	0.49, 0.82	*n* 10	0.70	0.56, 0.87	0.998
(10^9/L)	8	*n* 8	0.71	0.55, 0.92	*n* 10	0.63	0.51, 0.79	0.986
	16	*n* 8	0.65	0.50, 0.83	*n* 10	0.58*	0.46, 0.72	0.991
	24	*n* 8	0.66	0.51, 0.85	*n* 10	0.60	0.48, 0.75	0.996
	32	*n* 2	0.64	0.45, 0.92	*n* 10	0.60	0.48, 0.75	1.000
	40	*n* 8	0.58	0.45, 0.74	*n* 10	0.57*	0.46, 0.71	1.000
Granulocytes	Baseline	*n* 8	4.35	3.33, 5.69	*n* 10	4.76	3.74, 6.04	0.999
(10^9/L)	8	*n* 8	4.36	3.34, 5.70	*n* 10	4.48	3.52, 5.69	1.000
	16	*n* 8	4.02	3.08, 5.26	*n* 10	3.89	3.06, 4.95	1.000
	24	*n* 8	3.96	3.03, 5.18	*n* 10	4.31	3.39, 5.48	1.000
	32	*n* 2	3.75	2.53, 5.56	*n* 10	4.21	3.31, 5.35	0.999
	40	*n* 8	3.69	2.82, 4.82	*n* 10	3.63*	2.86, 4.61	1.000
Platelet count	Baseline	*n* 8	238	150, 327	*n* 10	208	129, 287	0.999
(10^9/L)	8	*n* 8	191	102, 279	*n* 10	264	185, 343	0.660**
	16	*n* 8	251	162, 339	*n* 10	234	155, 313	1.000
	24	*n* 8	198	109, 286	*n* 10	262	183, 341	0.801
	32	*n* 2	240	112, 367	*n* 10	268	188, 347	1.000
	40	*n* 8	203	114, 291	*n* 10	279*	200, 358	0.606**

* A significant difference from baseline within diet group ($p < 0.05$)

** A significant difference from baseline between diet groups ($p < 0.05$)
