# Supplementary material for: No Observed Adverse Effects on Health Were Detected in Adult Beagle Dogs When Fed a High-Calcium Diet for 40 Weeks
Source: Animals (Basel). 2021 Jun 16;11(6):1799. doi: 10.3390/ani11061799 (PMC8234157; doi:10.3390/ani11061799)
Supplement: Supplementary file 1 [file animals-11-01799-s001.zip › Table_S4.pdf]

Table S4. Diet digestibility, mineral excretion and balance

Additional biochemistry values given as means and 95% confidence intervals (CI) for control (*n* 8) and test (*n* 10) diet dog groups at baseline and at 8 week intervals. P values represent comparison between diet groups at the sample collection time point ($p < 0.05$ is statistically signficant).

	Weeks	Control			Test			*P*
			Mean	95% Cl		Mean	95% Cl	
Faecal calcium excretion (g/d)	Baseline	*n* 8	1.17	0.86, 1.59	*n* 10	1.21	0.92, 1.59	1.000
	8	*n* 8	2.20*	1.62, 2.99	*n* 10	6.75*	5.13, 8.89	<0.001**
	16	*n* 6	1.37	0.99, 1.90	*n* 10	6.62*	5.03, 8.71	<0.001**
	24	*n* 8	1.09	0.80, 1.48	*n* 10	6.28*	4.77, 8.26	<0.001**
	32	*n* 8	0.94	0.69, 1.28	*n* 10	6.22*	4.73, 8.19	<0.001**
	40	*n* 8	0.93	0.68, 1.26	*n* 10	5.86*	4.46, 7.72	<0.001**
Faecal phosphorus excretion (g/d)	Baseline	*n* 8	0.56	0.41, 0.75	*n* 10	0.59	0.45, 0.77	1.000
	8	*n* 8	1.06*	0.78, 1.43	*n* 10	3.44*	2.62, 4.51	<0.001**
	16	*n* 6	0.65	0.47, 0.90	*n* 10	3.44*	2.62, 4.51	<0.001**
	24	*n* 8	0.52	0.38, 0.70	*n* 10	3.26*	2.48, 4.28	<0.001**
	32	*n* 8	0.45	0.33, 0.61	*n* 10	3.21*	2.45, 4.21	<0.001**
	40	*n* 8	0.43	0.31, 0.58	*n* 10	2.97*	2.26, 3.90	<0.001**
Urinary calcium excretion (g/d)	Baseline	*n* 8	0.0019	0.0010, 0.0035	*n* 10	0.0033	0.0019, 0.0056	0.541
	8	*n* 8	0.0056*	0.0031, 0.0102	*n* 10	0.0039	0.0023, 0.0067	0.921**
	16	*n* 8	0.0052*	0.0028, 0.0094	*n* 10	0.0078*	0.0046, 0.0134	0.831
	24	*n* 8	0.0050*	0.0027, 0.0090	*n* 10	0.0068*	0.0040, 0.0116	0.967
	32	*n* 8	0.0031	0.0017, 0.0056	*n* 10	0.0075*	0.0044, 0.0129	0.036
	40	*n* 8	0.0042*	0.0023, 0.0077	*n* 10	0.0072*	0.0042, 0.0123	0.574
Urinary phosphate excretion (g/d)	Baseline	*n* 8	0.1429	0.0855, 0.2389	*n* 10	0.2641	0.1668, 0.4181	0.177
	8	*n* 8	0.3992*	0.2388, 0.6670	*n* 10	0.2538	0.1603, 0.4018	0.570**
	16	*n* 8	0.3081*	0.1843, 0.5148	*n* 10	0.6066*	0.3832, 0.9602	0.094
	24	*n* 8	0.3858*	0.2309, 0.6447	*n* 10	0.5994*	0.3786, 0.9488	0.606
	32	*n* 8	0.2383*	0.1426, 0.3982	*n* 10	0.5649*	0.3569, 0.8942	0.010
	40	*n* 8	0.3788*	0.2267, 0.6330	*n* 10	0.7344*	0.4639, 1.1625	0.111
Calcium digestibility (%)	Baseline	*n* 8	19.75	-13.16, 52.66	*n* 10	23.94	-5.50, 53.37	1.000
	8	*n* 8	-56.11*	-89.03, -23.20	*n* 10	24.81	-4.63, 54.24	<0.001**
	16	*n* 6	5.45	-41.10, 51.99	*n* 10	5.15	-24.29, 34.58	1.000
	24	*n* 8	7.47	-25.44, 40.38	*n* 10	28.65	-0.79, 58.08	0.875
	32	*n* 8	22.44	-10.48, 55.35	*n* 10	-6.47	-35.91, 22.97	0.538
	40	*n* 8	10.83	-22.09, 43.74	*n* 10	13.95	-15.49, 43.39	1.000
Phosphorus digestibility (%)	Baseline	*n* 8	31.66	14.69, 48.64	*n* 10	24.29	9.11, 39.48	0.991
	8	*n* 8	-45.65*	-62.63, -28.68	*n* 10	19.52	4.33, 34.70	<0.001**
	16	*n* 6	1.94*	-17.66, 21.53	*n* 10	5.36	-9.83, 20.54	1.000
	24	*n* 8	-1.98*	-18.95, 15.00	*n* 10	6.83	-8.35, 22.02	0.966
	32	*n* 8	14.62	-2.36, 31.59	*n* 10	0.88*	-14.30, 16.07	0.652
	40	*n* 8	-7.14*	-24.12, 9.83	*n* 10	0.68*	-14.51, 15.86	0.985
Calcium balance (g/d)	Baseline	*n* 8	0.27	-0.51, 1.05	*n* 10	0.37	-0.32, 1.07	1.000
	8	*n* 8	-0.81†	-1.59, -0.04	*n* 10	1.11†	0.41, 1.80	<0.001**
	16	*n* 6	0.02	-0.88, 0.91	*n* 10	0.89†	0.20, 1.59	0.308
	24	*n* 8	0.11	-0.66, 0.89	*n* 10	0.99†	0.29, 1.68	0.207
	32	*n* 8	0.23	-0.55, 1.00	*n* 10	0.24	-0.45, 0.93	1.000
	40	*n* 8	0.11	-0.66, 0.89	*n* 10	0.48	-0.21, 1.18	0.982
Phosphate balance (g/d)	Baseline	*n* 8	0.33	-0.14, 0.80	*n* 10	0.28	-0.14, 0.70	1.000
	8	*n* 8	-0.50*†	-0.97, -0.03	*n* 10	0.96*†	0.54, 1.38	<0.001**
	16	*n* 6	0.01	-0.53, 0.55	*n* 10	0.36	-0.06, 0.78	0.820
	24	*n* 8	-0.02	-0.49, 0.45	*n* 10	0.40	-0.02, 0.82	0.526
	32	*n* 8	0.13	-0.34, 0.60	*n* 10	0.03	-0.39, 0.45	1.000
	40	*n* 8	-0.06	-0.53, 0.41	*n* 10	0.03	-0.38, 0.45	1.000

* A significant difference from baseline within diet group *($p < 0.05$)*

** A significant difference from baseline between diet groups ($p < 0.05$)
† A significant difference from zero ($p < 0.05$)
